# Lower heart rate variability, an index of worse autonomic function, is associated with worse beta cell response to a glycemic load in vivo—The Maastricht Study

**DOI:** 10.1186/s12933-023-01837-0

**Published:** 2023-05-04

**Authors:** Elisabetta Rinaldi, Frank CT van der Heide, Enzo Bonora, Maddalena Trombetta, Chiara Zusi, Abraham A Kroon, Miranda T Schram, Carla JH van der Kallen, Anke Wesselius, Riccardo Bonadonna, Andrea Mari, Casper G Schalkwijk, Marleen MJ van Greevenbroek, Coen DA Stehouwer

**Affiliations:** 1 Division of Endocrinology, Diabetes and Metabolic Diseases, Department of Medicine, University of Verona, Policlinico GB Rossi, Piazzale L.A. Scuro 10, Verona, 37134 Italy; 2 CARIM School for Cardiovascular Diseases, Maastricht University (UM), Maastricht, the Netherlands; 3 Department of Internal Medicine, Maastricht University Medical Centre+ (MUMC+), Maastricht, the Netherlands; 4 Heart and Vascular Centre, MUMC+, Maastricht, the Netherlands; 5 Department of Epidemiology, UM, Maastricht, the Netherlands; 6 NUTRIM School for Nutrition and Translational Research in Metabolism, UM, Maastricht, the Netherlands; 7 Department of Medicine and Surgery, University of Parma, Parma, Italy; 8 Division of Endocrinology and Metabolic Diseases, Azienda Ospedaliera-Universitaria di Parma, Parma, Italy; 9 Institute of Neuroscience, National Research Council, Padua, Italy

## Abstract

**Objective:**

We investigated, using population-based data, whether worse autonomic function, estimated from lower 24-hour heart rate variability (HRV), was associated with beta cell function, assessed from beta cell response during an oral glucose tolerance test (OGTT).

**Research design and methods:**

We used cross-sectional data from The Maastricht Study, a population-based cohort study (N = 2,007; age, mean ± SD:60 ± 8 years; 52% men; and 24% with type 2 diabetes). We used linear regression analyses with adjustment for potential confounders (demographic, cardiovascular, and lifestyle factors) to study the associations of time- and frequency-domain HRV (composite scores) with overall beta cell response (estimated from a composite score calculated from: C-peptidogenic index, overall insulin secretion, beta cell glucose sensitivity, beta cell potentiation factor, and beta cell rate sensitivity). In addition, we tested for interaction by sex and glucose metabolism status.

**Results:**

After full adjustment, lower time- and frequency-domain HRV was significantly associated with lower overall beta cell response composite score (standardized beta, -0.055 [-0.098; -0.011] and − 0.051 [-0.095; -0.007], respectively). These associations were not modified by sex and there was no consistent pattern of interaction by glucose metabolism status.

**Conclusion:**

The present etiological study found that worse autonomic function, estimated from lower HRV, was associated with worse beta cell function, estimated from a composite score in a population-based sample which covered the entire spectrum of glucose metabolism. Hence, autonomic dysfunction may contribute to beta cell dysfunction and, ultimately, to the alteration of glucose metabolism status from normal glucose metabolism to prediabetes and type 2 diabetes.

**Supplementary Information:**

The online version contains supplementary material available at 10.1186/s12933-023-01837-0.

## Introduction

At present it remains incompletely understood whether autonomic dysfunction may contribute to the pathobiology of beta cell dysfunction, which plays a central role in the onset of prediabetes and type 2 diabetes. [1–3] Autonomic nerves are thought to contribute to the regulation of glucose homeostasis by transmitting information to beta cells on (1) an anticipated increase in glucose levels in the near future (i.e. during food intake) and (2) on prevailing central and peripheral glucose levels, initiating the secretion of, respectively, first- and second phase insulin secretion. [1–4] Biologically, parasympathetic nerves are thought to increase beta cell insulin secretion, whereas sympathetic nerves are thought to inhibit beta cell insulin secretion and stimulate alpha cell glucagon release. [1–3].

The recent development of non-invasive measurement techniques provides an opportunity to gain insight in whether worse autonomic function may contribute to beta cell function in vivo. [5] First, autonomic function can be non-invasively assessed as heart rate variability (HRV) from electrocardiograms (ECG), where lower HRV is thought to reflect worse autonomic function in the heart. [6] Biologically, lower HRV is thought to reflect relative overactivity of the sympathetic nerve system and relatively underactivity of the parasympathetic nerve system at the sinus node (i.e. a sympathetic-parasympathetic disbalance). [6] Second, beta cell response to a glycemic load can be estimated during an oral glucose tolerance test (OGTT) through both formula-based methods (C-peptidogenic index and overall insulin secretion) and through mathematical modeling (beta cell glucose sensitivity, beta cell potentiation factor, and beta cell rate sensitivity) [5, 7].

Current evidence has important limitations. The main limitation of current literature on the association of autonomic function, estimated from HRV, with beta cell function is that currently no population-based studies have yet investigated beta cell response to a glycemic load in vivo. However, there is some evidence. [8] Indeed, one previous population study found that lower HRV was associated with worse beta cell function during the fasting state (estimated from HOMA). [8].

In view of above, we investigated, using a large, well-characterized population-based cohort study, whether worse autonomic function, estimated from lower HRV, was associated with worse beta cell function, estimated as lower beta cell response during a 7-point OGTT. We hypothesized that lower HRV was associated with worse beta cell response to a glycemic load.

## Methods

### Study population and design

We used data from The Maastricht Study, a prospectively designed, population-based observational cohort study. The rationale and methodology have been described previously. [9] In brief, the study focuses on the etiology, pathophysiology, complications and comorbidities of type 2 diabetes mellitus and is characterized by an extensive phenotyping approach. Eligible for participation were all individuals aged between 40 and 75 years and living in the southern part of the Netherlands. Participants were recruited through mass media campaigns and from the municipal registries and the regional Diabetes Patient Registry via mailings. Recruitment was stratified according to known type 2 diabetes status, with an oversampling of individuals with type 2 diabetes, for reasons of efficiency. [9] The present report includes cross-sectional data of 3,451 participants who completed the baseline survey between November 2010 and September 2013. The examinations of each participant were performed within a time window of three months. The study has been approved by the institutional medical ethics committee (NL31329.068.10) and the Minister of Health, Welfare and Sports of the Netherlands (Permit 131088-105234-PG). All participants gave written informed consent. [9].

### Assessment of heart rate variability

Heart rate variability was assessed from ECGs, as reported previously. [10] All ECGs were recorded by use of a 12-lead Holter system (Fysiologic ECG Services, Amsterdam, the Netherlands) over a 24-h period. During recording time, participants were asked to follow their normal daily activities, except that they were asked not to take a shower or a bath. Recordings were analyzed with proprietary Holter Analysis Software at Fysiologic ECG Services (Fysiologic ECG Services, Amsterdam, the Netherlands) with an algorithm that excluded non-sinus cardiac cycles (e.g., artifacts and premature/ectopic beats), validated by manual inspection afterward. The software from Fysiologic ECG Services provided the intervals between the individual R waves of sinus beats (i.e. interbeat intervals, in milliseconds). From the obtained interbeat intervals, HRV was calculated by use of the publicly available free GNU Octave software, [11] according to the standard time- and frequency- domain measures defined by the (recently updated) recommendations of the Task Force document on HRV. [6, 12] After exclusion of non-sinus cardiac cycles, the minimum duration of the recording for HRV analysis was 18 h. We calculated the following time domain measures: the standard deviation (SD) of all normal-to-normal (NN) intervals (SDNN, in milliseconds [ms]); the SD of the averages of NN intervals in all 5-min segments of the entire recording (SDANN, in ms); the square root of the mean of the sum of squares of differences between adjacent NN intervals (RMSSD, in ms); the mean of the SDs of all NN intervals for all 5-min segments of the entire recording (SDNN index, in ms); the number of pairs of adjacent NN intervals differing by 50 ms in the entire recording (NN50 count, number); and the NN50 count divided by the total number of all NN intervals (pNN50, a percentage). Then, we calculated the following frequency domain measures using the Fast Fourier Transform: the variance of all NN intervals ≤ 0.4 Hz (total power [TP], in ms squared); power in the ultralow-frequency range (ULF, in ms squared; ≤0.003 Hz); power in the very-low-frequency range (VLF, in ms squared; 0.003–0.04 Hz); power in the low-frequency range (LF, in ms squared; 0.04–0.15 Hz); and the power in the high-frequency range (HF, in ms squared; 0.15–0.4 Hz). Individual *z*-scores were calculated for the time- and frequency- domain measures. We calculated the overall time- domain variable composite z-score using the following formula: ([SDNN + RMSSD + SDANN + SDNN index + pNN50]/5); and calculated overall frequency- domain variable using the following formula: ([TP + ULF + VLF + LF + HF]/5). Before we composed the composite z-score we checked whether associations of individual time- and frequency-domain HRV indices with individual measures of beta cell response were directionally consistent (in the complete study population), and this was the case.

### Beta cell response

After an overnight fast, all participants (except those who used insulin or had a fasting plasma glucose concentration above 11.0 mmol/L) underwent a standard 2-hour 75 g OGTT. Venous blood samples were collected before and at 15, 30, 45, 60, 90, and 120 min post oral glucose load intake. We estimated beta cell response using the following parameters: C-peptidogenic index _t0-30_, overall insulin secretion (C-peptide _AUC_/ glucose _AUC_ [CP_AUC_/G_AUC_]), beta cell glucose sensitivity, beta cell potentiation factor, and beta cell rate sensitivity. We used formula-based methods and mathematical modeling, as previously described. [5, 13] First, C-peptidogenic index _t0-30_, which reflects early insulin secretion after a glucose stimulus, was calculated as the difference in C-peptide level between baseline and 30 min post glucose load intake (ΔCP_30_) divided by the difference in glucose level between baseline and 30 min post glucose load intake (ΔG_30_) [5, 13]. Second, overall insulin secretion, an analogous index reflecting the overall OGTT response, was calculated as the area under the curve (AUC) of C-peptide divided by the AUC of glucose (CP_AUC_/G_AUC_) [5, 13]. To calculate AUC, C-peptide or glucose were plotted against time. Third, beta-cell function parameters were calculated using a previously developed model, [5] which describes the relationship between insulin secretion and glucose concentration by means of a dose-response function relating the two variables and an early secretion component. The dose-response is characterized by its average slope, termed glucose sensitivity, and early secretion by a parameter denoted as rate sensitivity, a marker of early phase insulin release. The dose-response function is modulated by a time-varying potentiation factor, which accounts for effects of sustained hyperglycemia and incretins. The potentiation factor excursion was calculated as the ratio between the values at the end of the 2-h OGTT and at baseline.

Next, we calculated an overall beta cell response composite score (“overall beta cell response”). We standardized individual beta cell response measures (i.e. expressed as z-scores) and calculated the overall beta cell response composite score as follows: (C-peptidogenic index _t0-30_ + overall insulin secretion + beta cell glucose sensitivity + beta cell potentiation factor + beta cell rate sensitivity)/5. Then, we re-standardized the composite score. Before we composed the composite z-score we checked whether associations of time- and frequency-domain HRV with individual measures of beta cell response were directionally consistent (in the complete study population), and this was the case.

### Covariates

As described previously, [9] we assessed educational level (low, intermediate, high), socio-economic status (income level and occupational status), smoking status (never, former, current), alcohol use (none, low, high), and history of cardiovascular disease by questionnaire; assessed total energy intake with a food frequency questionnaire; [14] assessed lipid-modifying, antihypertensive, and glucose-lowering medication use as part of a medication interview; assessed weight, height, and waist circumference during a physical examination; calculated body-mass index (BMI) based on body weight and height; measured office and 24-hour ambulatory blood pressure; measured total daily physical activity (hours/day) with an accelerometer; measured lipid profile in fasting venous plasma samples; estimated the Matsuda Index, an index of insulin sensitivity, using data from the OGTT; and calculated the estimated glomerular filtration rate (eGFR) based on serum creatinine and cystatin C.

### Statistical analysis

Characteristics of the study population were described as means and standard deviations (SD) for continuous variables or as number and proportions of participants per category for categorical variables (% of study population). We used multivariable linear regression analyses to investigate the associations of the independent variables (time- and frequency- domain HRV) with the dependent variables (overall beta cell response composite score; and C-peptidogenic index, overall insulin secretion, beta cell glucose sensitivity, beta cell potentiation factor, and beta cell rate sensitivity separately). Before we performed these analyses, we checked whether assumptions of linear regression were not violated and this was the case (we checked whether associations were linear, whether the residuals were normally distributed, and whether the homoscedasticity [homogenous distribution of variance] assumption was not violated). We checked these assumptions by visually inspecting relevant plots. These associations were presented as standardized betas with corresponding 95% confidence intervals [CI]). In addition, we inversed HRV so that associations were expressed per SD lower HRV (indicating progressively worse autonomic function). Last, we used complete case analysis.

We first analyzed associations without adjustment (crude model). In model 1, we adjusted for age, sex, and educational status (low, medium, high). We chose these variables because they are key demographic potential confounders. In model 2, we additionally adjusted for Matsuda Index, an index of insulin resistance. [7] We entered Matsuda Index in a separate model because beta-cell function is influenced by insulin resistance. In model 3, we additionally adjusted for cardiovascular risk factors and lifestyle factors. In model 3 A, we additionally adjusted for: BMI, total cholesterol / HDL cholesterol ratio, lipid-modifying medication (yes/no), smoking status (current, former, never), and alcohol consumption status (none, low, high). We adjusted for these potential confounders in a separate model as on one hand these factors may be confounders, but on the other hand these factors are also on the causal pathway, thus adjustment for these factors may be overadjustment (i.e. these factors can induce autonomic dysfunction which can lead to beta cell dysfunction). [15] In model 3B, we additionally adjusted for office systolic blood pressure and use of antihypertensive medication (yes/no). We adjusted for blood pressure in a separate model because blood pressure may be a confounder; a cause of autonomic dysfunction; and a descending proxy of autonomic function. [15, 16]

We tested for interaction by sex and glucose metabolism status to investigate whether associations under study differed by sex (i.e. between men and women) or glucose metabolism status (i.e. between individuals with type 2 diabetes, prediabetes, or normal glucose metabolism). We tested for interaction in the fully adjusted model by including interaction terms with the determinant (e.g. frequency-domain HRV*sex) and all covariates (e.g. age*sex), as previously described. [17].

To assess the robustness of our findings we performed a number of additional analyses. First, we analyzed the associations with the overall beta cell response composite score and individual beta cell response indices of individual measures of HRV (i.e. individual measures that were used to compose time and frequency domain composite scores). Second, and only for associations of time-and frequency-domain HRV with beta cell rate sensitivity as outcome, we performed logistic regression analyses in which beta cell rate sensitivity was categorized into tertiles (we performed this analysis for statistical reasons because the distribution of beta cell rate sensitivity was somewhat skewed and could not be normalized via logarithmical transformations). Third, we repeated the analyses with additional adjustment for total energy intake and physical activity. These potential confounders were not included in the main analyses because data were missing for a relatively large number of participants (up to n = 311 had missing data on one or more of these variables). Fourth, we additionally adjusted for kidney function (eGFR) and history of cardiovascular disease. We adjusted for these covariates in a separate model because they may be confounders but may also (in part) be mediators or descendants of the outcome. Fifth, we replaced waist circumference by BMI; educational status by occupational status or income level; and office systolic blood pressure by office diastolic blood pressure, systolic or diastolic 24-hour ambulatory blood pressure. Last, we analyzed the associations under study stratified by glucose metabolism status to check whether associations of time-and frequency-domain HRV with overall beta cell response were similar over the entire glucose metabolism spectrum (i.e. from normal glucose metabolism to prediabetes and type 2 diabetes).

All analyses were performed with Statistical Package for Social Sciences version 28.0 (IBM SPSS, IBM Corp, Armonk, NY, USA). For all analyses, a P-value < 0.05 was considered statistically significant.

## Results

### Selection and characteristics of the study population

**Fig. 1:**
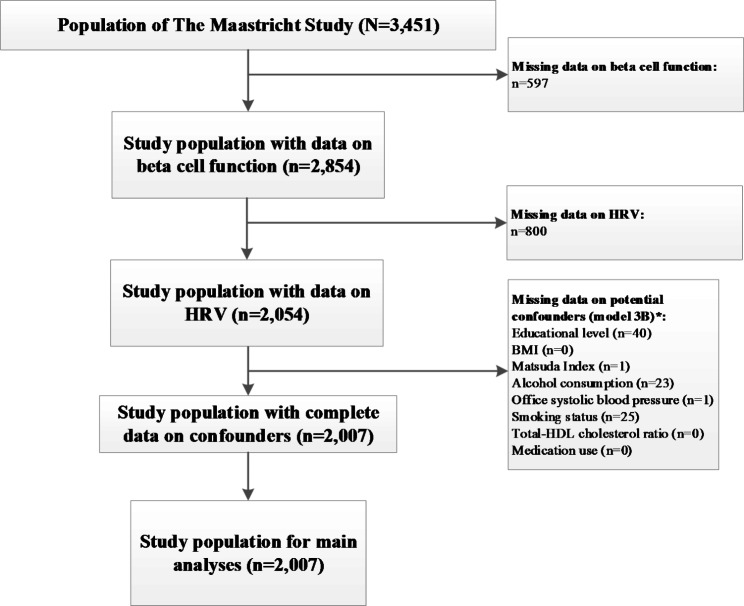
Study population selection * Not mutually exclusive Abbreviations: HRV, heart rate variability; HDL, high density lipoprotein; BMI, body-mass index

Figure 1 shows how the study population was arrived at. Complete data on HRV, outcomes and covariates were available in n = 2,007 participants.

Table 1 and Supplemental Table S1 show general participant characteristics according to tertiles of time- and frequency- domain HRV, respectively. The mean±SD age was 60 ±8 years; 52% were men; and 24% had type 2 diabetes. In general, individuals with a lower time- and frequency- domain HRV had a more adverse cardiovascular risk profile. General characteristics of participants included in the study were comparable to those of participants with missing data (Supplemental Table S2).

**Table 1:** General study population characteristics of the Maastricht Study according to tertiles of time-domain HRV

		HRV time-domain composite score			
Characteristic	Number of participants	Overall, N = 2,007	Tertile 1, N = 669	Tertile 2, N = 669	Tertile 3, N = 669
Age (years)	N = 2,007	59.88 ± 8.25	61.67 ± 7.47	60.11 ± 8.01	57.87 ± 8.76
Sex	N = 2,007				
Men		1,044 (52%)	326 (49%)	331 (49%)	387 (58%)
Women		963 (48%)	343 (51%)	338 (51%)	282 (42%)
Educational status	N = 2,007				
Low		637 (32%)	236 (35%)	230 (34%)	171 (26%)
Middle		551 (27%)	164 (25%)	178 (27%)	209 (31%)
High		819 (41%)	269 (40%)	261 (39%)	289 (43%)
Occupational status	N = 1,663				
Low		488 (29%)	166 (31%)	159 (29%)	163 (29%)
Middle		583 (35%)	186 (34%)	186 (33%)	211 (37%)
High		592 (36%)	188 (35%)	212 (38%)	192 (34%)
Income level (euros)	N = 1,554	2,066.08 ± 836.97	2,093.17 ± 881.22	2,083.99 ± 838.65	2,022.57 ± 790.26
Glucose metabolism status	N = 2,007				
Normal glucose metabolism		1,208 (60%)	330 (49%)	416 (62%)	462 (69%)
Prediabetes		323 (16%)	113 (17%)	119 (18%)	91 (14%)
Type 2 diabetes		476 (24%)	226 (34%)	134 (20%)	116 (17%)
Other types of diabetes		0 (0%)	0 (0%)	0 (0%)	0 (0%)
Glucose-lowering medication	N = 2,007	342 (17%)	172 (26%)	95 (14%)	75 (11%)
Matsuda Index (no unit)	N = 2,007	3.46 (2.04, 5.19)	2.92 (1.76, 4.51)	3.62 (2.14, 5.43)	3.93 (2.40, 5.85)
Office systolic blood pressure (mmHg)	N = 2,007	134.59 ± 17.94	136.39 ± 17.71	134.64 ± 18.05	132.75 ± 17.91
Office diastolic blood pressure (mmHg)	N = 2,007	76.61 ± 9.80	77.31 ± 9.88	76.88 ± 10.02	75.63 ± 9.43
24-hour ambulatory systolic blood pressure (mmHg)	N = 1,835	118.68 ± 11.35	119.54 ± 11.75	118.51 ± 10.99	117.99 ± 11.26
24-hour ambulatory diastolic blood pressure (mmHg)	N = 1,835	73.61 ± 7.01	74.00 ± 7.23	73.46 ± 6.87	73.36 ± 6.92
Use of antihypertensive medication	N = 2,007	744 (37%)	303 (45%)	224 (33%)	217 (32%)
Body-mass index (kg/m^2^)	N = 2,007	26.72 ± 4.23	27.42 ± 4.70	26.52 ± 3.98	26.23 ± 3.87
Waist circumference (cm)	N = 2,005	95.05 ± 12.81	97.10 ± 13.52	94.44 ± 12.36	93.61 ± 12.28
Alcohol consumption	N = 2,007				
None		335 (17%)	93 (14%)	114 (17%)	128 (19%)
Moderate		1,121 (56%)	350 (52%)	367 (55%)	404 (60%)
High		551 (27%)	226 (34%)	188 (28%)	137 (20%)
Total/HDL cholesterol ratio	N = 2,007	3.53 (2.87, 4.36)	3.56 (2.91, 4.40)	3.53 (2.87, 4.38)	3.47 (2.85, 4.29)
Use of lipid-modifying medication (yes/no)	N = 2,007	656 (33%)	271 (41%)	206 (31%)	179 (27%)
Smoking status	N = 2,007				
Never		687 (34%)	194 (29%)	238 (36%)	255 (38%)
Former		1,063 (53%)	383 (57%)	345 (52%)	335 (50%)
Current		257 (13%)	92 (14%)	86 (13%)	79 (12%)
Physical activity (hours/day)	N = 1,788	14.31 ± 8.08	13.83 ± 8.07	14.68 ± 8.38	14.42 ± 7.78
Total caloric intake (KJ/day)	N = 1,890	9,215.13 ± 2,553.35	9,095.53 ± 2,565.01	9,093.76 ± 2,408.70	9,456.67 ± 2,665.65
History of cardiovascular disease	N = 1,991	316 (16%)	126 (19%)	90 (14%)	100 (15%)
Estimated Glomerular Filtration Rate	N = 2,003	88.32 ± 14.31	87.37 ± 14.64	88.12 ± 14.24	89.47 ± 13.99
*HRV, Time-domain composite score (SD)*	N = 2,007	0.00 ± 1.00	-0.93 ± 0.30	-0.15 ± 0.21	1.07 ± 0.89
SDNN (ms)		135.54 ± 37.55	100.38 ± 17.52	134.89 ± 17.50	171.36 ± 33.14
SDANN (ms)		122.09 ± 36.13	90.63 ± 17.96	122.66 ± 19.76	152.97 ± 35.50
RMSSD (ms)		29.88 ± 17.98	18.99 ± 5.12	26.18 ± 6.79	44.48 ± 23.50
SDNN index (ms)		54.30 ± 18.31	39.32 ± 7.40	51.51 ± 7.60	72.07 ± 18.59
SDSD (ms)		29.88 ± 17.98	18.99 ± 5.12	26.18 ± 6.79	44.48 ± 23.50
pNN50 (%)		6.16 (2.66, 12.24)	2.19 (1.09, 3.91)	6.01 (3.85, 9.04)	15.14 (10.02, 24.16)
*HRV, Frequency-domain composite score (SD)*	N = 2,007	0.00 ± 1.00	-0.54 ± 0.28	-0.13 ± 0.28	0.67 ± 0.70
TP (ms [2])		11,589.40 (7,873.16, 16,499.85)	6,814.82 (5,047.99, 8,417.24)	12,083.60 (9,973.33, 14,188.90)	18,732.40 (14,987.20, 23,342.00)
ULF (ms [2])		9,840.92 (6,481.91, 13,973.95)	5,862.03 (4,170.16, 7,395.05)	10,429.70 (8,374.72, 12,625.10)	16,042.00 (11,901.40, 20,353.70)
VLF (ms [2])		1,075.88 (736.36, 1,556.57)	644.88 (489.58, 839.21)	1,073.34 (882.15, 1,312.46)	1,859.36 (1,410.70, 2,473.42)
LF (ms [2])		347.03 (207.59, 591.74)	192.20 (133.26, 274.39)	343.04 (244.30, 479.55)	691.66 (494.01, 966.11)
HF (ms [2])		84.26 (47.72, 147.19)	42.84 (29.88, 64.23)	83.69 (57.15, 121.17)	173.71 (109.62, 280.54)
*Indices of beta cell response*					
C-peptidogenic index (no unit)	N = 2,007	471.29 ± 1,003.20	413.50 ± 589.52	456.00 ± 1,204.82	544.37 ± 1,101.91
Overall insulin secretion (no unit)	N = 2,007	193.60 ± 79.46	189.74 ± 83.38	196.95 ± 79.61	194.11 ± 75.13
Βeta cell glucose sensitivity (pmol/min /m^2^/mM)	N = 2,007	82.76 ± 55.68	78.83 ± 57.67	84.55 ± 54.58	84.90 ± 54.59
Βeta cell potentiation factor (no unit)	N = 2,007	1.63 ± 0.69	1.53 ± 0.67	1.68 ± 0.73	1.68 ± 0.67
Βeta cell rate sensitivity (pmol/m^2^/mM)	N = 2,007	724.22 ± 942.79	655.40 ± 747.22	724.35 ± 863.60	792.90 ± 1,164.31

Table 1 shows general characteristics of the study population in the total study population and according to tertiles of time domain HRV. Data are presented as mean ± standard deviation, median (interquartile range) or number (%). Abbreviations: HbA1c, hemoglobin A1c; HDL, high-density lipoprotein; NGM, normal glucose metabolism; eGFR, estimated glomerular filtration rate; HRV, heart rate variability.

### Associations of time-domain HRV with beta cell response

**Fig. 2:**
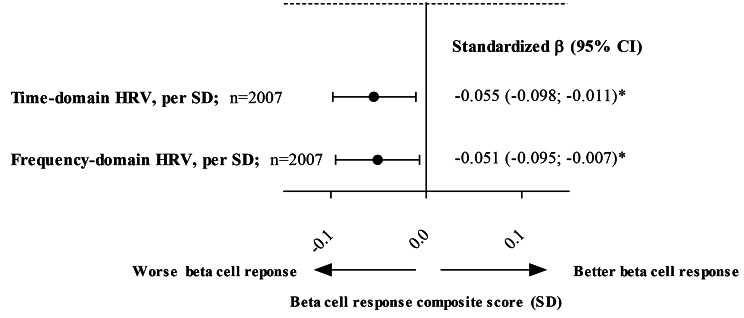
Figure 2 shows that lower HRV is significantly associated with lower overall beta cell response, estimated from the overall beta cell response composite score (in SD). Regression coefficients represent the difference in overall beta cell response composite score (in SD) per SD lower time-or frequency-domain HRV. Time domain HRV was estimated from SDNN, SDANN, RMSSD, SDNN index and pNN50; and frequency domain was estimated from TP, ULF, VLF, LF, and HF. Overall beta cell response composite score was estimated from C-peptidogenic index, overall insulin secretion, beta cell glucose sensitivity, beta cell potentiation factor, and beta cell rate sensitivity. Values per SD are reported in Table 1 Bold indicates P < 0.05 Variables entered in the models in addition to HRV: age, sex, educational status (low, medium, high), Matsuda Index, office systolic blood pressure, total cholesterol/HDL cholesterol ratio, use of antihypertensive or lipid-modifying medication (yes/no), BMI, smoking status (current, ever, never), and alcohol consumption status (none, low, high) Abbreviations: SD, standard deviation; CI, confidence interval; HRV, heart rate variability, HDL, high-density lipid; BMI, body-mass index. All abbreviations for indices of HRV are presented in the Methods section.

**Fig. 3:**
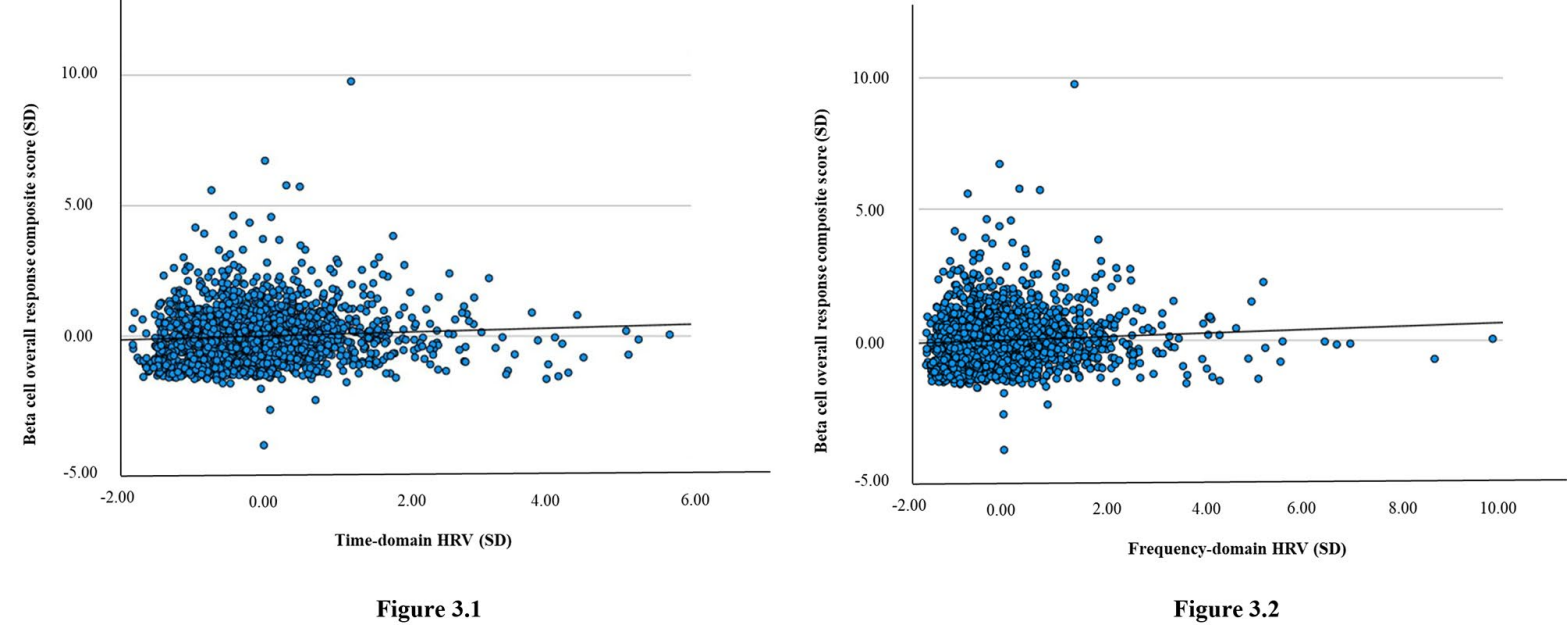
Figure 3.1 and 3.2, respectively, show the linear associations of lower time- and frequency-domain HRV (in SD) with lower overall beta cell response, assessed from the overall beta cell response composite score (in SD). Time domain HRV was estimated from SDNN, SDANN, RMSSD, SDNN index and pNN50; and frequency domain was estimated from TP, ULF, VLF, LF, and HF. Overall beta cell response composite score was estimated from C-peptidogenic index, overall insulin secretion, beta cell glucose sensitivity, beta cell potentiation factor, and beta cell rate sensitivity. Values per SD are reported in Table 1 Bold indicates P < 0.05 Abbreviations: SD, standard deviation; HRV, heart rate variability. All abbreviations for indices of HRV are presented in the Methods section.

After full adjustment (model 3B), lower time-domain HRV was significantly associated with lower overall beta cell response (one SD lower HRV was associated with 0.055 [95%CI: 0.011; 0.098] lower overall beta cell response composite score; Table 2; Fig. 2). Figure 3 shows the linear association of lower time-domain HRV with lower overall beta cell response.

**Table 2:** Associations of time- and frequency- domain heart rate variability with the beta cell composite score

		Beta cell composite score, per SD	
	Models	stβ [95% CI]	*P-value*
**HRV time- domain composite score, per SD lower**	Crude	**-0.070 (-0.114 to -0.026)**	**0.002**
	1	**-0.063 (-0.107 to -0.020)**	**0.004**
	2	**-0.067 (-0.111 to -0.023)**	**0.003**
	3 A	**-0.059 (-0.102 to -0.015)**	**0.008**
	3B	**-0.055 (-0.098 to -0.011)**	**0.013**
**HRV frequency- domain composite score, per SD lower**	Crude	**-0.073 (-0.116 to -0.029)**	**0.001**
	1	**-0.063 (-0.106 to -0.019)**	**0.005**
	2	**-0.066 (-0.110 to -0.022)**	**0.003**
	3 A	**-0.054 (-0.098 to -0.011)**	**0.015**
	3B	**-0.051 (-0.095 to -0.007)**	**0.022**

Standardized regression coefficient (stβ) represents the difference in beta cell composite score (in SD) per 1-SD lower time- or frequency-domain HRV composite score. Overall beta cell response composite score was estimated from C-peptidogenic index, overall insulin secretion, beta cell glucose sensitivity, beta cell potentiation factor, and beta cell rate sensitivity

Bold indicates p < 0.05

Variables entered in the models in addition to HRV: Crude: none; model 1: age, sex, and educational status (low, medium, high); model 2: model 1 + Matsuda Index; model 3 A: model 2 + total cholesterol/HDL cholesterol ratio, lipid-modifying medication (yes/no), BMI, smoking status (current, ever, never), and alcohol consumption status (none, low, high); Model 3B: model 3 A + office systolic blood pressure and use of antihypertensive medication

Abbreviations: SD, standard deviation; CI, confidence interval; HRV, heart rate variability, HDL, high density lipid; BMI, body-mass index. All abbreviations for indices of HRV are presented in the Methods section

After full adjustment (model 3B), lower time-domain HRV was significantly associated with lower C-peptidogenic index (per SD lower HRV 0.049 [95%CI: 0.003; 0.094] SD lower C-peptidogenic index); and in the same direction, although not statistically significantly, with lower overall insulin secretion (0.037 [0.006; 0.080]); lower beta cell glucose sensitivity (0.037 [0.007; 0.082]); lower beta cell potentiation factor (0.038 [0.006; 0.082]), and beta cell rate sensitivity (0.012 [0.033; 0.057]; Table 3).

**Table 3:** Associations of time- and frequency- domain heart rate variability with C-peptidogenic index, overall insulin secretion, beta cell glucose sensitivity, beta cell potentiation factor, and beta cell rate sensitivity

	C-peptidogenic index	*P-value*	Overall insulin secretion	*P-value*	Beta cell glucose sensitivity	*P-value*	Beta cell potentiation factor	*P-value*	Beta cell rate sensitivity	*P-value*
**HRV time- domain, per SD**	stβ [95% CI]		stβ [95% CI]		stβ [95% CI]		stβ [95% CI]		stβ [95% CI]	
Crude	**-0.061 (-0.104 to -0.017)**	**0.007**	-0.010 (-0.054 to 0.033)	0.641	-0.040 (-0.084 to 0.003)	0.070	**-0.073 (-0.116 to -0.029)**	**0.001**	-0.038 (-0.082 to 0.006)	0.088
1	**-0.060 (-0.104 to -0.016)**	**0.008**	-0.004 (-0.048 to 0.040)	0.870	-0.043 (-0.086 to 0.001)	0.058	**-0.073 (-0.117 to -0.029)**	**0.001**	-0.021 (-0.065 to 0.023)	0.340
2	**-0.059 (-0.104 to -0.014)**	**0.010**	-0.037 (-0.081 to 0.006)	0.089	**-0.048 (-0.093 to -0.004)**	**0.033**	**-0.045 (-0.088 to -0.001**)	**0.043**	-0.023 (-0.067 to 0.022)	0.315
3 A	**-0.051 (-0.097 to -0.006)**	**0.027**	-0.041 (-0.084 to 0.002)	0.060	-0.040 (-0.084 to 0.004)	0.077	-0.038 (-0.081 to 0.006)	0.089	-0.016 (-0.061 to 0.029)	0.49
3B	**-0.049 (-0.094 to -0.003)**	**0.035**	-0.037 (-0.080 to 0.006)	0.092	-0.037 (-0.082 to 0.007)	0.098	-0.038 (-0.082 to 0.006)	0.087	-0.012 (-0.057 to 0.033)	0.589
**HRV frequency- domain, per SD**	stβ [95% CI]		stβ [95% CI]		stβ [95% CI]		stβ [95% CI]		stβ [95% CI]	
Crude	**-0.061 (-0.105 to -0.018)**	**0.006**	-0.014 (-0.058 to 0.030)	0.770	**-0.044 (-0.088 to -0.0001)**	**0.049**	**-0.073 (-0.116 to -0.029)**	**0.001**	-0.038 (-0.082 to 0.005)	0.086
1	**-0.060 (-0.104 to -0.015)**	**0.009**	-0.005 (-0.049 to 0.040)	0.833	**-0.045 (-0.090 to -0.001)**	**0.044**	**-0.072 (-0.116 to -0.028)**	**0.001**	-0.017 (-0.062 to 0.027)	0.443
2	**-0.059 (-0.104 to -0.014)**	**0.011**	-0.036 (-0.080 to 0.007)	0.100	**-0.051 (-0.095 to -0.006)**	**0.026**	**-0.046 (-0.089 to -0.002)**	**0.041**	-0.018 (-0.063 to 0.026)	0.417
3 A	**-0.050 (-0.096 to -0.004)**	**0.033**	-0.039 (-0.082 to 0.005)	0.082	-0.039 (-0.084 to 0.006)	0.087	-0.035 (-0.079 to 0.009)	0.118	-0.010 (-0.056 to 0.035)	0.652
3B	**-0.048 (-0.094 to -0.002)**	**0.040**	-0.035 (-0.078 to 0.009)	0.117	-0.036 (-0.081 to 0.008)	0.109	-0.035 (-0.078 to 0.012)	0.122	-0.008 (-0.053 to 0.037)	0.734

Standardized regression coefficient (stβ) represents the difference in beta cell index (in SD) per 1-SD lower HRV measure, where time domain HRV was estimated from SDNN, SDANN, RMSSD, SDNN index and pNN50; and frequency domain was estimated from TP, ULF, VLF, LF, and HF. Overall beta cell response composite score was estimated from C-peptidogenic index, overall insulin secretion, beta cell glucose sensitivity, beta cell potentiation factor, and beta cell rate sensitivity

Bold indicates p < 0.05

Variables entered in the models in addition to HRV: Crude: none; model 1: age, sex, and educational status (low, medium, high); model 2: model 1 + Matsuda Index; model 3 A: model 2 + total cholesterol/HDL cholesterol ratio, lipid-modifying medication (yes/no), BMI, smoking status (current, ever, never), and alcohol consumption status (none, low, high); Model 3B: model 3 A + office systolic blood pressure and use of antihypertensive medication

Abbreviations: SD, standard deviation; CI, confidence interval; HRV, heart rate variability, HDL, high density lipid; BMI, body-mass index. All abbreviations for indices of HRV are presented in the Methods section

### Associations of frequency-domain HRV with beta cell response

After full adjustment (model 3B), lower frequency-domain HRV was significantly associated with lower overall beta cell response (one SD lower HRV was associated with 0.051 [95%CI: 0.007; 0.095] lower overall beta cell response composite score; Table 2; Fig. 2). Figure 3 shows the linear association of lower frequency-domain HRV with lower overall beta cell response.

After full adjustment (model 3B), lower frequency-domain HRV was significantly associated with lower C-peptidogenic index (per SD lower HRV 0.048 [95%CI: 0.002; 0.094] SD lower C-peptidogenic index); and in the same direction, though not statistically significantly, with lower overall insulin secretion (0.035 [-0.009; 0.078]); lower beta cell glucose sensitivity (0.036 [-0.008; 0.081]); lower beta cell potentiation factor (0.035 [-0.012; 0.078]) and lower beta cell rate sensitivity (0.008 [0.037; -0.053]; Table 3).

### Interaction analyses

Sex and type 2 diabetes status did not modify any association; however, prediabetes status did (all P-values for interaction are shown in Supplemental Table S3). Prediabetes status modified the associations of time- and frequency- domain with beta cell potentiation factor. Analyses stratified by glucose metabolism status did not show a consistent pattern (Supplemental Table S3).

### Additional analyses

Quantitatively similar results were observed in a range of additional analyses. First, we had quantitatively similar findings when we analyzed associations with the overall beta cell response composite score and individual beta cell response indices of individual measures of HRV that were used to compose the time- and frequency- domain composite scores (Supplemental Tables S4 and S5). Second, we had similar findings when we investigated the associations with beta cell rate sensitivity as outcome when beta cell rate sensitivity was categorized in tertiles (instead of as a continuous variable; Supplemental Table S6). Third, associations did not materially change when we additionally adjusted the associations under study for energy intake and physical activity; or for eGFR and history of cardiovascular disease (Supplemental Tables S7 and S8). Fourth, associations did not materially change when we replaced BMI by waist circumference; educational status by occupational status or income level; or office systolic blood pressure by office diastolic blood pressure, systolic or diastolic 24-hour ambulatory blood pressure (Supplemental Tables S9 and S10). Last, in glucose metabolism status-stratified analyses we had the following findings: 1) we found that in individuals with type 2 diabetes the betas for all analyses were directionally similar to the analyses shown in Tables 2 and 3; 2) we found that in individuals with normal glucose metabolism only the beta of the association of HRV with C-peptidogenic index was directionally similar to the betas shown in Tables 2 and 3 (betas were approximately null for other indices); and 3) we found, unexpectedly, that in individuals with prediabetes the betas for most associations were directionally opposite to the betas shown in Tables 2 and 3 (Supplemental Tables S11 and S12). To further examine these unexpected findings in prediabetes we performed additional analyses. In these analyses we replaced Matsuda index with total insulin secretion during the oral glucose tolerance test [assessed as C-peptide _AUC_]). We performed this additional analysis because insulin is an important regulator of autonomic function [5] and levels of insulin are substantially higher in individuals with prediabetes than in individuals with type 2 diabetes and normal glucose metabolism. [5] After adjustment for insulin levels we found that associations were substantially attenuated and not statistically significant, except beta cell potentiation factor (Supplemental Table S13).

## Discussion

The present population-based study has three main findings. First, lower HRV was significantly associated with lower overall beta cell response, estimated from a composite score. Second, we had numerically similar findings when we analyzed associations of HRV with all individual beta cell response indices (i.e. C-peptidogenic index, overall insulin secretion, beta cell glucose sensitivity, and beta cell glucose sensitivity) except for beta cell rate sensitivity. Third, in glucose metabolism status-stratified analyses, we found that lower HRV was associated with lower C-peptidogenic index in individuals with type 2 diabetes and in individuals with normal glucose metabolism, but not in individuals with prediabetes.

This study is the first study to demonstrate that worse autonomic function is associated with worse in vivo beta cell response to a glycemic load at population level. Our findings expand on knowledge acquired in previous population-based studies [8, 18] which reported that worse autonomic function, estimated from lower HRV, was associated with worse beta cell function in the fasting state (estimated from HOMA) [8] and with a higher incidence of type 2 diabetes. [18] In addition, evidence from many experimental animal data are in agreement with our findings. [19].

Our findings are in support of the concept that autonomic function may contribute to beta cell response to a glycemic load. [1–3, 19] Biologically, an imbalance in sympathetic and parasympathetic nerve activity (i.e. overactive sympathetic nerves and underactive parasympathetic nerves) may cause a lower beta cell response to a glycemic load because the parasympathetic nervous system stimulates insulin secretion whereas the sympathetic nervous system exerts an opposite activity. [1–3, 19] Mechanistically, in response to an increase in glucose levels, which is sensed in the pancreas, peripheral organs, and the brain, parasympathetic nerves endings are thought release acetylcholine, which can increase insulin secretion. [1–3, 19]

In our study the association between HRV and beta cell rate sensitivity (an index of first phase insulin secretion) was the weakest and almost absent. [4] This is not consistent with available literature in which autonomic system seems to influence the first phase of insulin secretion. [1–4] A possible explanation is that HRV does not adequately reflect autonomic dysfunction during first phase of insulin secretion. [4] HRV reflects the imbalance between parasympathetic and sympathetic nerve system. However, the imbalance may not adequately reflect autonomic dysfunction during the first phase of insulin secretion as sympathetic nerves may not contribute to the first phase of insulin secretion. [4] Indeed, previous studies have found that sympathetic nerves inhibit insulin secretion during the hypoglycemic state, but not during the first phase of insulin secretion. [4].

Our findings are consistent with the concept that autonomic dysfunction may contribute to beta cell dysfunction before the onset of type 2 diabetes. Indeed, we found that lower HRV was associated with lower C-peptidogenic not only in individuals with type 2 diabetes but also in individuals with normal glucose metabolism.

However, in individuals with prediabetes lower HRV was directionally associated with a *greater* C-peptidogenic index. A possible explanation for the latter finding may be that in our model we did not account for all factors that contribute to the regulation of beta cell function. [5] Indeed, in line with this concept, we found in additional analyses that the association of lower HRV with greater C-peptidogenic index was considerably attenuated (and was almost null) after adjustment for insulin levels during the OGTT. In line with this, we found that insulin levels during an OGTT are substantially higher in prediabetes than in type 2 diabetes and normal glucose metabolism (also in the present dataset [data not shown]). [5] Additionally, we consider that findings in prediabetes may also reflect measurement error and/or the play of chance. [20] Findings for prediabetes in this study population may be relatively more prone to such phenomena as the sample size of prediabetes was relatively the smallest (in comparison with type 2 diabetes and normal glucose metabolism).

Our findings may have implications for future clinical research that aims to prevent beta cell dysfunction. Future studies may investigate whether early prevention of autonomic dysfunction, may reduce the rate of decline of beta cell function and, ultimately, may contribute to the prevention and/or slowing of the onset of type 2 diabetes. [21] Indeed, we previously demonstrated a linear trend between deterioration of glucose metabolism status and lower HRV. [14] Such prevention may be possible via the early modification of risk factors for neurodegeneration (e.g. early prevention of hyperglycemia and high alcohol consumption). [21, 22] Of note, however, although such a therapy may contribute, the effects of such an approach may be modest given weak betas we identified. In addition, it is important to take potential sex differences into account when designing such therapies. Given that there are presently few human data available on sex differences in the association between autonomic function and beta cell function, [8, 18] we warrant it important that future studies investigate whether sex differences may exist.

Strengths of this study are (1) the large size of this population-based cohort with oversampling of individuals with type 2 diabetes, which enabled accurate investigation of the associations under study over the entire spectrum of glucose metabolism (i.e. from normal glucose metabolism to prediabetes and type 2 diabetes); (2) the extensive number of potential confounders that were considered; (3) the use of state-of-the-art and novel methods (e.g. HRV) to assess all variables included in this study; and (4) the considerable number of additional analyses, which generally yielded consistent findings. [20].

The study has certain limitations. First, due to the cross-sectional nature of the study, causal inferences should be made with caution. [23] There may be a bidirectional relationship between autonomic dysfunction and worse beta cell response to glycemia and both processes may induce a vicious circle. [21] On the one hand, autonomic dysfunction may lead to higher levels of glycemia, which are detrimental for beta cells, resulting in a worse beta cell response to glycemia. [21] On the other hand, beta cell dysfunction may lead to higher glucose levels, which is detrimental for autonomic nerves. [21] Second, we may have underestimated the strength of the associations under study if such associations were similar or stronger in participants that were excluded from the study population (who generally tend to be less healthy; e.g. individuals were not eligible for an OGTT if they used insulin or had a fasting plasma glucose of 11.0 mmol/L).^24^ Such range restriction may lead to underestimated associations. [24] Third, although we took an extensive set of confounders into account, we cannot fully exclude bias due to unmeasured confounding (e.g., environmental factors such as air pollution). [25] Last, we studied Caucasian individuals aged 40–75 years with access to high-quality diabetes care. Therefore, the generalizability of our results to other populations requires further study.

## Conclusions

The present etiological study found that worse autonomic function, estimated from lower HRV, was cross-sectionally associated with worse beta cell function, estimated from a composite score, in a population-based sample which covered the entire spectrum of glucose metabolism. Hence, autonomic dysfunction may contribute to beta cell dysfunction and, ultimately, to the alteration of glucose metabolism status from normal glucose metabolism to prediabetes and type 2 diabetes.

## Electronic supplementary material

Below is the link to the electronic supplementary material.

Supplementary Material 1

## Data Availability

Data are available from The Maastricht Study for any researcher who meets the criteria for access to confidential data; the corresponding author may be contacted to request data.
