# Supplementary material for: Lower heart rate variability, an index of worse autonomic function, is associated with worse beta cell response to a glycemic load in vivo—The Maastricht Study
Source: Cardiovasc Diabetol. 2023 May 4;22:105. doi: 10.1186/s12933-023-01837-0 (PMC10161476; doi:10.1186/s12933-023-01837-0)
Supplement: Supplementary file 1 — Supplementary Material 1 [file 12933_2023_1837_MOESM1_ESM.docx]

### **Supplemental Material**

**Content**

- Supplemental Table S1 General study population characteristics according to tertiles of frequency-domain heart rate variability
- Supplemental Table S2 General study population characteristics of the included and excluded participants
- Supplemental Table S3 P-values of interaction terms with glucose metabolism status and sex in the associations of time- and frequency-domain HRV with indices of beta cell response
- Supplemental Table S4 Associations of individual time- and frequency- domain HRV indices with the overall beta cell response composite score (model 3B)
- Supplemental Table S5 Associations of individual time- and frequency- domain HRV indices with C-peptidogenic index, overall insulin secretion, beta cell glucose sensitivity, beta cell potentiation factor, and beta cell rate sensitivity (model 3B)
- Supplemental Table S6 Associations of time- and frequency-domain HRV with beta cell rate sensitivity, where rate sensitivity was categorized in tertiles
- Supplemental Table S7 Associations of time- and frequency-domain HRV with the overall beta cell response composite score, after additional adjustment for total caloric intake and physical activity (model 4), kidney function (eGFR; model 5), or history of CVD (model 6)
- Supplemental Table S8 Associations of time- and frequency-domain HRV with C-peptidogenic index, overall insulin secretion, beta cell glucose sensitivity, beta cell potentiation factor, and beta cell rate sensitivity after additional adjustment for total caloric intake and physical activity (model 4), kidney function (eGFR; model 5), or history of CVD (model 6)
- Supplemental Table S9 Associations of time- and frequency-domain HRV with the overall beta cell response composite score, where BMI was replaced with waist circumference (model 4A); educational level was replaced with occupational status (model 4B) or income level (model 4C); and office systolic blood pressure was replaced with office diastolic blood pressure (model 4D), 24-hour ambulatory systolic blood pressure (model 4E), or 24-hour ambulatory diastolic blood pressure (model 4F)
- Supplemental Table S10 Associations of time- and frequency-domain HRV with C-peptidogenic index, overall insulin secretion, beta cell glucose sensitivity, beta cell potentiation factor, and beta cell rate sensitivity where BMI was replaced with waist circumference (model 4A); educational level was replaced with occupational status (model 4B) or income level (model 4C); and office systolic blood pressure was replaced with office diastolic blood pressure (model 4D), 24-hour ambulatory systolic blood pressure (model 4E), or 24-hour ambulatory diastolic blood pressure (model 4F)
- Supplemental Table S11 Glucose metabolism status-stratified associations of time- and frequency- domain heart rate variability with C-peptidogenic index, overall insulin secretion, beta cell glucose sensitivity, beta cell potentiation factor, and beta cell rate sensitivity (model 3B)
- Supplemental Table S12 Glucose metabolism status-stratified associations of time- and frequency- domain heart rate variability with the beta cell composite score (model 3B)
- Supplemental Table S13 Associations of time- and frequency- domain heart rate variability with C-peptidogenic index, overall insulin secretion, beta cell glucose sensitivity, beta cell potentiation factor, and beta cell rate sensitivity in individuals with prediabetes, with adjustment for Matsuda Index (model 2A) or adjustment for total insulin secretion, estimated as C-peptide AUC from the 7-point oral glucose tolerance test (model 2B)

| Supplemental Table S1 General study population characteristics according to tertiles of frequency-domain HRV | | | | | | | |
| --- | --- | --- | --- | --- | --- | --- | --- |
|  | | **HRV frequency-domain composite score** | | | | | |
| Characteristic | Number of participants | Overall, N = 2,007 | Tertile 1, N=669 | Tertile 2, N=669 | Tertile 3, N=669 | | |
| Age (years) | N=2,007 | 59.88± 8.25 | 62.11± 7.29 | 60.16± 8.05 | 57.38± 8.65 | | |
| Sex | N=2,007 |  |  |  |  | | |
| Men |  | 1,044 (52%) | 323 (48%) | 332 (50%) | 389 (58%) | | |
| Women |  | 963 (48%) | 346 (52%) | 337 (50%) | 280 (42%) | | |
| Educational status | N=2,007 |  |  |  |  | | |
| Low |  | 637 (32%) | 242 (36%) | 234 (35%) | 161 (24%) | | |
| Middle |  | 551 (27%) | 162 (24%) | 168 (25%) | 221 (33%) | | |
| High |  | 819 (41%) | 265 (40%) | 267 (40%) | 287 (43%) | | |
| Occupational level | N=1,663 |  |  |  |  | | |
| Low |  | 488 (29%) | 158 (29%) | 172 (32%) | 158 (28%) | | |
| Middle |  | 583 (35%) | 197 (36%) | 179 (33%) | 207 (37%) | | |
| High |  | 592 (36%) | 200 (33%) | 194 (36%) | 198 (35%) | | |
| Income level (euros) | N=1,554 | 2,066.08 ± 836.97 | 2,085.92 ± 883.16 | 2,082.45 ± 837.32 | 2,031.61 ± 790.60 | | |
| Glucose metabolism status | N=2,007 |  |  |  |  | | |
| Normal glucose metabolism |  | 1,208 (60%) | 321 (48%) | 425 (62%) | 462 (69%) | | |
| Prediabetes |  | 323 (16%) | 124 (19%) | 111 (15%) | 102 (15%) | | |
| Type 2 diabetes |  | 476 (24%) | 224 (34%) | 142 (22%) | 105 (16%) |  |  |
| Other types of diabetes |  | 0 (0%) | 0 (0%) | 0 (0%) | 0 (0%) | |  |
| Glucose-lowering medication | N=2,007 | 342 (17%) | 169 (25%) | 102 (15%) | 71 (11%) | |  |
| Matsuda Index | N=2,007 | 3.46 (2.04, 5.19) | 2.88 (1.74, 4.46) | 3.59 (2.18, 5.45) | 3.98 (2.42, 5.82) | |  |
| Office systolic blood pressure (mmHg) | N=2,007 | 134.59 ± 17.94 | 136.91 ± 17.75 | 134.40 ± 18.42 | 132.48 ± 17.39 | |  |
| Office diastolic blood pressure (mmHg) | N=2,007 | 76.61 ± 9.80 | 77.38 ± 9.77 | 76.66 ± 10.09 | 75.79 ± 9.48 | |  |
| 24-hour ambulatory systolic blood pressure (mmHg) | N=1,835 | 118.68 ± 11.35 | 119.76 ± 11.82 | 118.24 ± 11.05 | 118.04 ± 11.10 | |  |
| 24-hour ambulatory diastolic blood pressure (mmHg) | N=1,835 | 73.61 ± 7.01 | 73.92 ± 7.31 | 73.33 ± 6.81 | 73.57 ± 6.90 | |  |
| Use of antihypertensive medication (yes/no) | N=2,007 | 744 (37%) | 319 (48%) | 229 (34%) | 196 (29%) | |  |
| Body-mass index (kg/m2) | N=2,007 | 26.72 ± 4.23 | 27.52 ± 4.68 | 26.54 ± 4.10 | 26.11 ± 3.73 | |  |
| Waist circumference (cm) | N=2,005 | 95.05 ± 12.81 | 97.44 ± 13.62 | 94.31 ± 12.34 | 93.40 ± 12.09 | |  |
| Alcohol consumption | N=2,007 |  |  |  |  | |  |
| None |  | 335 (17%) | 103 (15%) | 105 (16%) | 127 (19%) | |  |
| Moderate |  | 1,121 (56%) | 342 (51%) | 372 (56%) | 407 (61%) | | |
| High |  | 551 (27%) | 224 (33%) | 192 (29%) | 135 (20%) | | |
| Total/HDL cholesterol ratio | N=2,007 | 3.53 (2.87, 4.36) | 3.56 (2.89, 4.40) | 3.50 (2.85, 4.38) | 3.50 (2.89, 4.29) | | |
| Use of lipid-modifying medication (yes/no) | N=2,007 | 656 (33%) | 279 (42%) | 202 (30%) | 175 (26%) | | |
| Smoking status | N=2,007 |  |  |  |  | | |
| Never |  | 687 (34%) | 191 (29%) | 236 (35%) | 260 (39%) | | |
| Former |  | 1,063 (53%) | 379 (57%) | 346 (52%) | 338 (51%) | | |
| Current |  | 257 (13%) | 99 (15%) | 87 (13%) | 71 (11%) | | |
| Physical activity (hours/day) | N=1,788 | 14.31 ± 8.08 | 13.74 ± 7.90 | 14.91 ± 8.40 | 14.29 ± 7.91 | | |
| Total caloric intake (KJ/day) | N=1,890 | 9,215.13 ± 2,553.35 | 9,052.41 ± 2,564.97 | 9,115.10 ± 2,401.69 | 9,480.60 ± 2,670.02 | | |
| History of cardiovascular disease | N=1,991 | 316 (16%) | 128 (19%) | 100 (15%) | 88 (13%) | | |
| Estimated Glomerular Filtration Rate | N=2,003 | 88.32 ± 14.31 | 86.64 ± 14.84 | 88.34 ± 13.89 | 89.98 ± 14.00 | | |
| **Determinants** |  |  |  |  |  | | |
| *HRV, Time-domain composite score (SD)* | N=2,007 | 0.00 ± 1.00 | -0.69 ± 0.33 | -0.11 ± 0.35 | 0.80 ± 0.82 | | |
| SDNN (ms) |  | 135.54 ± 37.55 | 100.24 ± 17.41 | 134.10 ± 17.07 | 172.29 ± 32.21 | | |
| SDANN (ms) |  | 122.09 ± 36.13 | 90.55 ± 17.90 | 121.48 ± 19.09 | 154.23 ± 34.69 | | |
| RMSSD (ms) |  | 29.88 ± 17.98 | 19.51 ± 6.01 | 27.10 ± 8.78 | 43.04 ± 23.85 | | |
| SDNN index (ms) |  | 54.30 ± 18.31 | 39.07 ± 7.16 | 51.64 ± 7.23 | 72.18 ± 18.54 | | |
| SDSD (ms) |  | 29.88 ± 17.98 | 19.51 ± 6.01 | 27.10 ± 8.78 | 43.04 ± 23.85 | | |
| pNN50 (%) |  | 6.16 (2.66, 12.24) | 2.26 (1.11, 4.03) | 6.16 (3.81, 9.73) | 13.97 (9.04, 22.85) | | |
| *HRV, Frequency-domain composite score (SD)* | N=2,007 | 0.00 ± 1.00 | -0.61 ± 0.17 | -0.13 ± 0.14 | 0.73 ± 0.64 | | |
| TP (ms^2^) |  | 11,589.40 (7,873.16, 16,499.85) | 6,792.09 (5,018.46, 8,410.98) | 11,958.30 (9,837.80, 13,882.00) | 18,924.20 (15,496.70, 23,474.60) | | |
| ULF (ms^2^) |  | 9,840.92 (6,481.91, 13,973.95) | 5,850.61 (4,165.43, 7,360.11) | 10,304.80 (8,061.78, 12,509.10) | 16,284.00 (12,436.30, 20,458.90) | | |
| VLF (ms^2^) |  | 1,075.88 (736.36, 1,556.57) | 633.38 (485.93, 810.70) | 1,071.67 (884.47, 1,283.38) | 1,864.39 (1,428.75, 2,473.42) | | |
| LF (ms^2^) |  | 347.03 (207.59, 591.74) | 189.50 (132.12, 257.22) | 344.69 (262.36, 471.07) | 712.31 (513.60, 975.19) | | |
| HF (ms^2^) |  | 84.26 (47.72, 147.19) | 43.44 (30.16, 65.20) | 86.59 (57.68, 127.73) | 162.02 (102.58, 275.97) | | |
| **Outcomes** |  |  |  |  |  | | |
| C-peptidogenic index (no unit) | N=2,007 | 471.29 ± 1,003.20 | 412.39 ± 613.12 | 444.55 ± 1,171.54 | 556.92 ± 1,123.52 | | |
| Overall Insulin secretion (no unit) | N=2,007 | 193.60 ± 79.46 | 190.45 ± 82.62 | 195.56 ± 80.55 | 194.78 ± 75.05 | | |
| Beta cell glucose sensitivity (pmol/min/m^2^/mM) | N=2,007 | 82.76 ± 55.68 | 78.38 ± 56.74 | 83.75 ± 54.61 | 86.16 ± 55.46 | | |
| Beta cell potentiation factor (no unit) | N=2,007 | 1.63 ± 0.69 | 1.55 ± 0.67 | 1.64 ± 0.72 | 1.70 ± 0.68 | | |
| Beta cell rate sensitivity (pmol/m^2^/mM) | N=2,007 | 724.22 ± 942.79 | 655.25 ± 759.59 | 715.78 ± 820.58 | 801.61 ± 1,186.62 | | |

Data are presented as mean ± standard deviation, median (interquartile range) or number (%).

Abbreviations: HbA1c, hemoglobin A1c; HDL, high-density lipoprotein; NGM, normal glucose metabolism; eGFR, estimated glomerular filtration rate; HRV, heart rate variability.

Supplemental Table S2 General study population characteristics of the included and excluded participants

| Characteristic | Included, N=2,007 | Number of in/excluded participants with missing data | Excluded, N=1,444 |
| --- | --- | --- | --- |
| Age (years) | 59.88± 8.25 | 0/0 | 59.60± 8.30 |
| Sex |  | 0/0 |  |
| Men | 1,044 (52%) |  | 731 (51%) |
| Women | 963 (48%) |  | 713 (49%) |
| Educational status |  | 0/74 |  |
| Low | 637 (32%) |  | 498 (36%) |
| Middle | 551 (27%) |  | 403 (29%) |
| high | 819 (41%) |  | 469 (34%) |
| Occupational status |  | 344/283 |  |
| Low | 488 (29%) |  | 405 (35%) |
| Middle | 583 (35%) |  | 412 (36%) |
| High | 592 (36%) |  | 344 (30%) |
| Income level (euros) | 2,066.08 ± 836.97 | 453/413 | 1,934.71 ± 786.12 |
| Glucose metabolism status |  | 0/0 |  |
| Normal glucose metabolism status | 1,208 (60%) |  | 716 (50%) |
| Prediabetes | 323 (16%) |  | 188 (13%) |
| Type 2 diabetes | 476 (24%) |  | 499 (35%) |
| Other types of diabetes | 0 (0%) | 0/41 | 41 (2%) |
| Glucose-lowering medication | 342 (17%) | 0/0 | 465 (32%) |
| Matsuda Index (no unit) | 3.46 (2.04, 5.19) | 0/523 | 3.49 (2.01, 5.39) |
| Office systolic blood pressure (mmHg) | 134.59 ± 17.94 | 0/2 | 135.75 ± 18.57 |
| Office diastolic blood pressure (mmHg) | 76.61 ± 9.80 | 0/2 | 75.55 ± 9.90 |
| 24-hour ambulatory systolic blood pressure (mmHg) | 118.68 ± 11.35 | 172/275 | 119.38 ± 12.18 |
| 24-hour ambulatory diastolic blood pressure (mmHg) | 73.61 ± 7.01 | 172/275 | 72.91 ± 7.08 |
| Use of antihypertensive medication (yes/no) | 744 (37%) | 0/0 | 648 (45%) |
| Body-mass index (kg/m^2^) | 26.72 ± 4.23 | 0/3 | 27.60 ± 4.94 |
| waist circumference (cm) | 95.05 ± 12.81 | 2/2 | 97.21 ± 14.99 |
| Alcohol consumption |  | 0/48 |  |
| None | 335 (17%) |  | 301 (22%) |
| Moderate | 1,121 (56%) |  | 767 (55%) |
| High | 551 (27%) |  | 328 (23%) |
| Total/HDL cholesterol ratio | 3.53 (2.87, 4.36) | 0/4 | 3.38 (2.75, 4.20) |
| Use of lipid-modifying medication (yes/no) | 656 (33%) | 0/0 | 603 (42%) |
| Smoking status |  | 0/50 |  |
| Never | 687 (34%) |  | 485 (35%) |
| Former | 1,063 (53%) |  | 696 (50%) |
| Current | 257 (13%) |  | 213 (15%) |
| Physical activity (hours/day) | 14.31 ± 8.08 | 219/220 | 13.67 ± 8.17 |
| Total caloric intake (KJ/day) | 9,215.13 ± 2,553.35 | 117/108 | 9,053.90 ± 2,516.81 |
| History of cardiovascular disease | 316 (16%) | 16/51 | 252 (18%) |
| Estimated Glomerular Filtration Rate | 88.32 ± 14.31 | 4/29 | 87.84 ± 15.80 |
| SDNN (ms) | 135.54 ± 37.55 | 0/987 | 124.04 ± 37.99 |
| SDANN (ms) | 122.09 ± 36.13 | 0/987 | 111.83 ± 36.62 |
| RMSSD (ms) | 29.88 ± 17.98 | 0/987 | 29.01 ± 17.58 |
| SDNN index (ms) | 54.30 ± 18.31 | 0/987 | 49.78 ± 17.87 |
| pNN50 (%) | 6.16 (2.66, 12.24) | 0/987 | 5.66 (2.14, 12.53) |
| TP (ms^2^) | 11,589.40 (7,873.16, 16,499.85) | 0/987 | 9,570.26 (6,238.30, 14,006.20) |
| ULF (ms^2^) | 9,840.92 (6,481.91, 13,973.95) | 0/987 | 8,154.15 (5,049.28, 12,047.40) |
| VLF (ms^2^) | 1,075.88 (736.36, 1,556.57) | 0/987 | 896.47 (580.99, 1,380.89) |
| LF (ms^2^) | 347.03 (207.59, 591.74) | 0/987 | 290.82 (162.51, 499.96) |
| HF (ms^2^) | 84.26 (47.72, 147.19) | 0/987 | 73.61 (38.95, 139.65) |
| C-peptidogenic index (no unit) | 471.29 ± 1,003.20 | 0/526 | 441.40 ± 1,268.47 |
| Overall Insulin secretion (no unit) | 193.60 ± 79.46 | 0/522 | 197.94 ± 81.10 |
| Beta cell glucose sensitivity (pmol/min/m^2^/mM) | 82.76 ± 55.68 | 0/462 | 81.90 ± 54.30 |
| Beta cell potentiation factor (no unit) | 1.63 ± 0.69 | 0/462 | 1.63 ± 0.66 |
| Beta cell rate sensitivity (pmol/m^2^/mM) | 724.22 ± 942.79 | 0/462 | 798.46 ± 1,031.84 |

Data are presented as mean ± standard deviation, median (interquartile range) or number (%).

Abbreviations: BMI, body-mass index; HbA1c, hemoglobin A1c; HDL, high-density lipoprotein; NGM, normal glucose metabolism; eGFR, estimated glomerular filtration rate. All abbreviations for indices of HRV are presented in the Methods section.

Supplemental Table S3 P-values of interaction terms with glucose metabolism status and sex in the associations of time-and frequency-domain HRV with indices of beta cell response

|  |  | **Prediabetes** | **Type 2 diabetes** | **Sex** |
| --- | --- | --- | --- | --- |
|  | N | P-value for interaction | P-value for interaction | P-value for interaction |
| **HRV time-domain** | |  |  |  |
| Beta cell response composite score | 2,007 | 0.66 | 0.50 | 0.77 |
| C-peptidogenic index | 2,007 | 0.23 | 0.80 | 0.13 |
| Overall insulin secretion | 2,007 | 0.36 | 0.14 | 0.26 |
| Beta cell glucose sensitivity | 2,007 | 0.31 | 0.55 | 0.51 |
| Beta cell potentiation factor | 2,007 | **0.01*** | 0.85 | 0.97 |
| Beta cell rate sensitivity | 2,007 | 0.34 | 0.46 | 0.50 |
| **HRV frequency-domain** |  |  |  |  |
| Beta cell response composite score | 2,007 | 0.57 | 0.61 | 0.71 |
| C-peptidogenic index | 2,007 | 0.29 | 0.52 | 0.10 |
| Overall insulin secretion | 2,007 | 0.23 | 0.52 | 0.21 |
| Beta cell glucose sensitivity | 2,007 | 0.26 | 0.42 | 0.47 |
| Beta cell potentiation factor | 2,007 | **0.01**** | 0.73 | 0.68 |
| Rate sensitivity | 2,007 | 0.30 | 0.67 | 0.62 |

Supplemental Table S3 shows P-values for interaction terms that were calculated for glucose metabolism status (i.e., prediabetes versus normal glucose metabolism status and type 2 diabetes versus normal glucose metabolism status) or sex with the main determinants (i.e. time- or frequency-domain HRV score). P-values were calculated in the fully adjusted models. Time-domain HRV was estimated from SDNN, SDANN, RMSSD, SDNN index and pNN50; and frequency-domain was estimated from TP, ULF, VLF, LF, and HF.

* After stratification by glucose metabolism status, in individuals with prediabetes lower time-domain HRV was more strongly associated with lower beta cell potentiation factor (model 3B, per SD lower time-domain HRV 0.18 [95% CI: 0.06 to 0.29] SD lower potentiation factor) than in individuals with normal glucose metabolism (model 3B, per SD lower time-domain HRV 0.01 [95% CI: -0.05 to 0.07] SD *higher* potentiation factor) and in individuals with type 2 diabetes (model 3B, per SD lower time-domain HRV 0.03 [95% CI: -0.06 to 0.12] SD lower potentiation factor).

** After stratification by glucose metabolism status, in individuals with prediabetes lower frequency-domain HRV was more strongly associated with lower beta cell potentiation factor (model 3B, per SD lower frequency-domain HRV 0.17 [95% CI: 0.05 to 0.28] SD lower potentiation factor) than in individuals with normal glucose metabolism (model 3B, per SD lower frequency-domain HRV 0.00 [95% CI: -0.05 to 0.05] SD lower potentiation factor) and in individuals with type 2 diabetes (model 3B, per SD lower frequency-domain HRV 0.02 [95% CI: -0.07 to 0.11] SD lower potentiation factor).

Variables in models are interaction terms with sex or glucose metabolism status of the following variables: time-domain HRV or frequency-domain HRV, age, sex, educational status, Matsuda Index, office systolic blood pressure, total cholesterol/HDL cholesterol ratio, use of antihypertensive or lipid-modifying medication, BMI, smoking status, and alcohol consumption status.

P-value < 0.05 denotes statistically significant interaction.

Abbreviations: HRV, heart rate variability, HDL, high density lipid; BMI, body-mass index. All abbreviations for indices of HRV are presented in the Methods section.

Supplemental Table S4 Associations of individual time-and frequency-domain HRV indices with the overall beta cell response composite score (model 3B)

|  | Beta cell response composite score |  |
| --- | --- | --- |
| **HRV time-domain, per SD** | stβ [95% CI] | *P-value* |
| SDNN, per SD lower | **-0.06 (-0.10 to -0.02)** | **0.007** |
| RMSSD, per SD lower | -0.03 (-0.07 to 0.02) | 0.247 |
| SDANN, per SD lower | **-0.06 (-0.19 to -0.01)** | **0.010** |
| SDNN index, per SD lower | **-0.05 (-0.10 to -0.01)** | **0.024** |
| pNN50, per SD lower | -0.03 (-0.08 to 0.01) | 0.123 |
| **HRV frequency-domain, per SD** | stβ [95% CI] |  |
| TP, per SD lower | **-0.06 (-0.10 to -0.01)** | **0.012** |
| ULF, per SD lower | **-0.055 (-0.10 to -0.01)** | **0.013** |
| VLF, per SD lower | -0.04 (-0.08 to 0.01) | 0.100 |
| LF, per SD lower | -0.02 (-0.06 to 0.02) | 0.382 |
| HF, per SD lower | -0.04 (-0.08 to 0.01) | 0.084 |

Standardized regression coefficient (stβ) represents the difference in overall beta cell response composite score (in SD) per SD lower SDNN, SDANN, RMSSD, SDNN index and pNN50, TP, ULF, VLF, LF, and HF.

Bold indicates P-value<0.05.

Variables entered in the models in addition to HRV: age, sex, and educational status (low, medium, high), Matsuda Index, office systolic blood pressure, total cholesterol/HDL cholesterol ratio, use of antihypertensive or lipid-modifying medication (yes/no), BMI, smoking status (current, ever, never), and alcohol consumption status (none, low, high).

Abbreviations: SD, standard deviation; CI, confidence interval; HRV, heart rate variability, HDL, high density lipid; BMI, body-mass index. All abbreviations for indices of HRV are presented in the Methods section.

Supplemental Table S5 Associations of individual time-and frequency-domain HRV indices with C-peptidogenic index, overall insulin secretion, beta cell glucose sensitivity, beta cell potentiation, beta cell rate sensitivity (model 3B)

|  | C-peptidogenic index |  | Overall insulin secretion |  | Beta cell glucose sensitivity |  | Beta cell potentiation factor |  | | Beta cell rate sensitivity | |  |  |
| --- | --- | --- | --- | --- | --- | --- | --- | --- | --- | --- | --- | --- | --- |
| **HRV time-domain, per SD** | stβ [95% CI] | *P-value* | stβ [95% CI] | *P-value* | stβ [95% CI] | *P-value* | stβ [95% CI] | *P-value* | | stβ [95% CI] | | *P-value* |  |
| SDNN, per SD lower | -0.06 (-0.12 to -0.01) | **0.025** | -0.05 (-0.10 to 0.00) | 0.054 | -0.05 (-0.09 to 0.00) | **0.047** | -0.02 (-0.07 to 0.04) | 0.53 | | -0.01 (-0.06 to 0.05) | | 0.77 |  |
| RMSSD, per SD lower | -0.03 (-0.07 to 0.02) | 0.21 | -0.02 (-0.06 to 0.02) | 0.33 | -0.02 (-0.06 to 0.02) | 0.33 | -0.01 (-0.05 to 0.03) | 0.60 | | 0.00 (-0.04 to 0.05) | | 0.90 |  |
| SDANN, per SD lower | -0.05 (-0.09 to 0.00) | **0.044** | -0.04 (-0.08 to 0.01) | 0.10 | -0.03 (-0.08 to 0.01) | 0.13 | -0.04 (-0.09 to 0.00) | 0.060 | | -0.02 (-0.07 to 0.02) | | 0.36 |  |
| SDNN index, per SD lower | -0.05 (-0.09 to 0.00) | **0.044** | -0.04 (-0.08 to 0.01) | 0.10 | -0.03 (-0.08 to 0.01) | 0.13 | -0.04 (-0.09 to 0.00) | 0.060 | | -0.02 (-0.07 to 0.02) | | 0.36 |  |
| pNN50, per SD lower | -0.04 (-0.08 to 0.01) | 0.11 | -0.03 (-0.07 to 0.02) | 0.24 | -0.03 (-0.07 to 0.01) | 0.19 | -0.02 (-0.06 to 0.03) | 0.47 | | 0.00 (-0.04 to 0.04) | | 0.98 |  |
| **HRV frequency-domain, per SD** | stβ [95% CI] |  | stβ [95% CI] |  | stβ [95% CI] |  | stβ [95% CI] |  | | stβ [95% CI] | |  |  |
| TP, per SD lower | -0.07 (-0.12 to -0.01) | **0.015** | -0.05 (-0.10 to 0.01) | 0.084 | -0.04 (-0.09 to 0.00) | 0.063 | -0.02 (-0.07 to 0.04) | 0.55 | 0.00 (-0.06 to 0.05) | | 0.86 | | |
| ULF, per SD lower | -0.05 (-0.09 to 0.00) | **0.040** | -0.03 (-0.08 to 0.01) | 0.13 | -0.04 (-0.08 to 0.01) | 0.11 | -0.04 (-0.09 to 0.00) | **0.043** | | -0.01 (-0.06 to 0.03) | | 0.54 |  |
| VLF, per SD lower | -0.04 (-0.09 to 0.00) | 0.069 | -0.02 (-0.07 to 0.02) | 0.30 | -0.03 (-0.07 to 0.02) | 0.27 | -0.02 (-0.07 to 0.02) | 0.32 | | 0.00 (-0.05 to 0.04) | | 0.90 |  |
| LF, per SD lower | -0.02 (-0.07 to 0.02) | 0.36 | -0.02 (-0.06 to 0.03) | 0.41 | -0.03 (-0.07 to 0.02) | 0.22 | -0.01 (-0.05 to 0.04) | 0.78 | | 0.01 (-0.03 to 0.06) | | 0.62 |  |
| HF, per SD lower | -0.03 (-0.08 to 0.01) | 0.16 | -0.03 (-0.07 to 0.01) | 0.14 | -0.02 (-0.06 to 0.02) | 0.34 | -0.02 (-0.06 to 0.02) | 0.32 | | -0.01 (-0.06 to 0.03) | | 0.57 |  |

Standardized regression coefficient (stβ) represents the difference in beta cell index (in SD) per SD lower SDNN, SDANN, RMSSD, SDNN index and pNN50, TP, ULF, VLF, LF, and HF. Values per SD are reported in Table 1.

Bold indicates P-value<0.05.

Variables entered in the models in addition to HRV: age, sex, and educational status (low, medium, high), Matsuda Index, office systolic blood pressure, total cholesterol/HDL cholesterol ratio, use of antihypertensive or lipid-modifying medication (yes/no), BMI, smoking status (current, ever, never), and alcohol consumption status (none, low, high).

Abbreviations: SD, standard deviation; CI, confidence interval; HRV, heart rate variability, HDL, high density lipid; BMI, body-mass index. All abbreviations for indices of HRV are presented in the Methods section.

Supplemental Table S6 Associations of time-and frequency-domain HRV with beta cell rate sensitivity, where beta cell rate sensitivity was categorized in tertiles

|  | High versus low beta cell rate sensitivity | *P-value* | Middle versus low beta cell rate sensitivity | *P-value* |
| --- | --- | --- | --- | --- |
| **HRV time-domain, per SD** | OR [95% CI] |  | OR [95% CI] |  |
| Model Crude, n=2,007 | 1.08 (0.97 to 1.21) | 0.14 | 0.99 (0.90 to 1.12) | 0.99 |
| Model 1, n=2,007 | 1.05 (0.94 to 1.17) | 0.41 | 0.99 (0.88 to 1.10) | 0.81 |
| Model 2, n=2,007 | 1.04 (0.93 to 1.17) | 0.45 | 0.96 (0.86 to 1.08) | 0.52 |
| Model 3A, n=2,007 | 1.04 (0.93 to 1.17) | 0.49 | 0.96 (0.86 to 1.08) | 0.50 |
| Model 3B, n=2,007 | 1.03 (0.92 to 1.16) | 0.60 | 0.95 (0.85 to 1.07) | 0.41 |
| **HRV frequency-domain, per SD** | OR [95% CI] |  | OR [95% CI] |  |
| Model Crude, n=2,007 | 1.00 (0.90 to 1.12) | 0.97 | 0.94 (0.84 to 1.04) | 0.94 |
| Model 1, n=2,007 | 1.02 (0.91 to 1.14) | 0.70 | 0.98 (0.88 to 1.09) | 0.72 |
| Model 2, n=2,007 | 1.05 (0.93 to 1.17) | 0.44 | 0.98 (0.88 to 1.10) | 0.77 |
| Model 3A, n=2,007 | 1.06 (0.94 to 1.12) | 0.35 | 1.00 (0.89 to 1.12) | 0.94 |
| Model 3B, n=2,007 | 1.07 (0.95 to 1.20) | 0.28 | 1.00 (0.90 to 1.13) | 0.95 |

Regression coefficient represents the odds for middle or high versus low beta cell rate sensitivity per SD lower time- or frequency- domain HRV. Values per SD are numerically identical to the numbers reported in Table 1.

Bold indicates P-value<0.05.

Variables entered in the models in addition to HRV: Crude: none (crude results); model 1: age, sex, and educational status (low, medium, high); model 2: model 1 + Matsuda Index; model 3A: model 2 + total cholesterol/HDL cholesterol ratio, use or lipid-modifying medication (yes/no), BMI, smoking status (current, ever, never), and alcohol consumption status (none, low, high); model 3B: model 3A + office systolic blood pressure and use of antihypertensive medication.

Abbreviations: OR, odds ratio; SD, standard deviation; CI, confidence interval; HRV, heart rate variability, HDL, high density lipid; BMI, body-mass index. All abbreviations for indices of HRV are presented in the Methods section.

Supplemental Table S7 Associations of time- and frequency-domain HRV with the overall beta cell response composite score, after additional adjustment for total caloric intake and physical activity (model 4), kidney function (eGFR; model 5), or history of CVD (model 6)

|  | Beta cell response composite score |  |
| --- | --- | --- |
| **HRV time-domain, per SD** | stβ [95% CI] | *P-value* |
| Model 4, n=1,699 | **-0.06 (-0.10 to -0.01)** | **0.024** |
| Model 5, n=2,003 | **-0.05 (-0.09 to -0.01)** | **0.028** |
| Model 6, n=1,991 | **-0.05 (-0.10 to -0.01)** | **0.018** |
| **HRV frequency-domain, per SD** | stβ [95% CI] |  |
| Model 4, n=1,699 | **-0.05 (-0.10 to -0.002)** | **0.041** |
| Model 5, n=2,003 | **-0.05 (-0.09 to -0.001)** | **0.044** |
| Model 6, n=1,991 | **-0.04 (-0.09 to -0.006)** | **0.027** |

Standardized regression coefficient (stβ) represents the difference in overall beta cell response composite score in SD, per SD lower HRV composite score index.

Bold indicates P-value<0.05.

Variables entered in the models in addition to HRV: age, sex, and educational status (low, medium, high), Matsuda Index, office systolic blood pressure, total cholesterol/HDL cholesterol ratio, use of antihypertensive or lipid-modifying medication (yes/no), BMI, smoking status (current, ever, never), and alcohol consumption status (none, low, high).

Abbreviations: SD, standard deviation; CI, confidence interval; HRV, heart rate variability, HDL, high density lipid; BMI, body-mass index.

Supplemental Table S8 Associations of time- and frequency- domain HRV with C-peptidogenic index, overall insulin secretion, beta cell glucose sensitivity, beta cell potentiation, and beta cell rate sensitivity after additional adjustment for total caloric intake and physical activity (model 4), kidney function (eGFR; model 5), or history of CVD (model 6)

|  | C-peptidogenic index | *P-value* | Overall insulin secretion | *P-value* | Beta cell glucose sensitivity | *P-value* | Beta cell potentiation factor | *P-value* | Beta cell rate sensitivity | *P-value* |
| --- | --- | --- | --- | --- | --- | --- | --- | --- | --- | --- |
| **HRV time-domain, per SD** | stβ [95% CI] |  | stβ [95% CI] |  | stβ [95% CI] |  | stβ [95% CI] |  | stβ [95% CI] |  |
| Model 4, n=1,699 | -0.06 (-0.11 to -0.01) | **0.026** | -0.04 (-0.08 to 0.01) | 0.13 | -0.04 (-0.09 to 0.01) | 0.089 | -0.03 (-0.08 to 0.02) | 0.24 | -0.01 (-0.06 to 0.04) | 0.71 |
| Model 5, n=2,003 | -0.05 (-0.09 to 0.00) | **0.047** | -0.03 (-0.07 to 0.02) | 0.21 | -0.03 (-0.08 to 0.01) | 0.15 | -0.03 (-0.08 to 0.01) | 0.13 | -0.01 (-0.06 to 0.03) | 0.55 |
| Model 6, n=1,991 | -0.05 (-0.09 to 0.00) | **0.039** | -0.03 (-0.08 to 0.01) | 0.13 | -0.04 (-0.08 to 0.01) | 0.11 | -0.04 (-0.08 to 0.01) | 0.10 | -0.01 (-0.06 to 0.03) | 0.63 |
| **HRV frequency-domain, per SD** | stβ [95% CI] |  | stβ [95% CI] |  | stβ [95% CI] |  | stβ [95% CI] |  | stβ [95% CI] |  |
| Model 4, n=1,699 | -0.05 (-0.10 to 0.00) | **0.032** | -0.03 (-0.08 to 0.02) | 0.19 | -0.04 (-0.09 to 0.01) | 0.11 | -0.03 (-0.08 to 0.02) | 0.26 | 0.00 (-0.05 to 0.05) | 0.92 |
| Model 5, n=2,003 | -0.05 (-0.09 to 0.00) | 0.054 | -0.03 (-0.07 to 0.02) | 0.25 | -0.03 (-0.08 to 0.01) | 0.16 | -0.03 (-0.07 to 0.01) | 0.17 | -0.01 (-0.05 to 0.04) | 0.69 |
| Model 6 n=1,991 | -0.05 (-0.09 to 0.00) | **0.045** | -0.03 (-0.08 to 0.01) | 0.14 | -0.04 (-0.08 to 0.01) | 0.11 | -0.03 (-0.08 to 0.01) | 0.13 | -0.01 (-0.05 to 0.04) | 0.77 |

Standardized regression coefficient (stβ) represents the difference in beta cell index in SD, per SD lower HRV composite score index. Values per SD are numerically similar to the numbers reported in Table 1.

Bold indicates P-value<0.05.

Variables entered in the models in addition to HRV: age, sex, and educational status (low, medium, high), Matsuda Index, office systolic blood pressure, total cholesterol/HDL cholesterol ratio, use of antihypertensive or lipid-modifying medication (yes/no), BMI, smoking status (current, ever, never), and alcohol consumption status (none, low, high).

Abbreviations: SD, standard deviation; CI, confidence interval; HRV, heart rate variability, HDL, high density lipid; BMI, body-mass index. All abbreviations for indices of HRV are presented in the Methods section.

Supplemental Table S9 Associations of time- and frequency-domain HRV with the overall beta cell response composite score, where BMI was replaced with waist circumference (model 4A); educational level was replaced with occupational status (model 4B) or income level (model 4C); and office systolic blood pressure was replaced with office diastolic blood pressure (model 4D), 24-hour ambulatory systolic blood pressure (model 4E), or 24-hour ambulatory diastolic blood pressure (model 4F)

|  | Beta cell response composite score |  |
| --- | --- | --- |
| **HRV time-domain, per SD** | stβ [95% CI] | *P-value* |
| Model 4A, n=2,005 | **-0.05 (-0.10 to -0.01)** | **0.014** |
| Model 4B, n=1,676 | **-0.06 (-0.10 to -0.01)** | **0.022** |
| Model 4C, n=1,440 | -0.05 (-0.10 to 0.001) | 0.056 |
| Model 4D, n=2,007 | **-0.06 (-0.10 to -0.01)** | **0.012** |
| Model 4E. n=1,836 | **-0.06 (-0.11 to -0.01)** | **0.011** |
| Model 4F, n=1,836 | **-0.06 (-0.11 to -0.02)** | **0.007** |
| **HRV frequency-domain, per SD** | stβ [95% CI] |  |
| Model 4A, n=2,005 | **-0.05 (-0.094 to -0.01)** | **0.024** |
| Model 4B, n=1,676 | **-0.05 (-0.10 to -0.01)** | **0.032** |
| Model 4C, n=1,440 | -0.05 (-0.10 to 0.004) | 0.072 |
| Model 4D, n=2,007 | **-0.05 (-0.10 to -0.01)** | **0.021** |
| Model 4E. n=1,836 | **-0.06 (-0.10 to -0.01)** | **0.017** |
| Model 4F, n=1,836 | **-0.06 (-0.11 to -0.01)** | **0.011** |

Standardized regression coefficient (stβ) represents the difference in overall beta cell response composite score in SD per SD lower HRV index.

Bold indicates P-value<0.05.

Variables entered in the models in addition to HRV are (where appropriate): age, sex, and educational status (low, medium, high), Matsuda Index, office systolic blood pressure, total cholesterol/HDL cholesterol ratio, use of antihypertensive or lipid-modifying medication (yes/no), BMI, smoking status (current, ever, never), and alcohol consumption status (none, low, high).

Abbreviations: SD, standard deviation; CI, confidence interval; HRV, heart rate variability, HDL, high density lipid; BMI, body-mass index. All abbreviations for indices of HRV are presented in the Methods section.

Supplemental Table S10 Associations of time-and frequency-domain HRV with C-peptidogenic index, overall insulin secretion, beta cell glucose sensitivity, beta cell potentiation factor and beta cell rate sensitivity, where BMI was replaced with waist circumference (model 3A); educational level was replaced with occupational status (model 3B) or income level (model 3C); and office systolic blood pressure was replaced with office diastolic blood pressure (model 3D), 24-hour ambulatory systolic blood pressure (model 3E), or 24-hour ambulatory diastolic blood pressure (model 3F)

|  | C-peptidogenic index | *P-value* | Overall insulin secretion | *P-value* | Beta cell glucose sensitivity | *P-value* | Beta cell potentiation factor | *P-value* | Beta cell rate sensitivity | *P-value* |
| --- | --- | --- | --- | --- | --- | --- | --- | --- | --- | --- |
| **HRV time-domain, per SD** | stβ [95% CI] |  | stβ [95% CI] |  | stβ [95% CI] |  | stβ [95% CI] |  | stβ [95% CI] |  |
| Model 4A, n=2,005 | **-0.05 (-0.09 to 0.00)** | **0.035** | -0.04 (-0.08 to 0.01) | 0.09 | -0.04 (-0.08 to 0.01) | 0.10 | -0.04 (-0.08 to 0.01) | 0.10 | -0.01 (-0.06 to 0.03) | 0.59 |
| Model 4B, n=1,676 | **-0.06 (-0.11 to -0.01)** | **0.030** | -0.04 (-0.09 to 0.01) | 0.11 | -0.03 (-0.08 to 0.02) | 0.18 | -0.04 (-0.09 to 0.01) | 0.12 | -0.01 (-0.06 to 0.04) | 0.65 |
| Model 4C, n=1,440 | **-0.06 (-0.12 to -0.01)** | **0.025** | -0.05 (-0.10 to 0.00) | 0.054 | **-0.05 (-0.09 to 0.00)** | **0.047** | -0.02 (-0.07 to 0.04) | 0.53 | -0.01 (-0.06 to 0.05) | 0.77 |
| Model 4D, n=2,007 | **-0.05 (-0.10 to 0.00)** | **0.034** | -0.04 (-0.08 to 0.00) | 0.078 | -0.04 (-0.08 to 0.00) | 0.081 | -0.04 (-0.08 to 0.01) | 0.10 | -0.01 (-0.06 to 0.03) | 0.60 |
| Model 4E. n=1,836 | **-0.05 (-0.10 to 0.00)** | **0.050** | -0.04 (-0.09 to 0.00) | 0.064 | -0.04 (-0.09 to 0.00) | 0.059 | -0.04 (-0.09 to 0.01) | 0.085 | -0.01 (-0.06 to 0.03) | 0.55 |
| Model 4F, n=1,836 | **-0.05 (-0.10 to 0.00)** | **0.045** | **-0.05 (-0.09 to 0.00)** | **0.043** | **-0.05 (-0.09 to 0.00)** | **0.047** | -0.04 (-0.09 to 0.00) | 0.080 | -0.02 (-0.06 to 0.03) | 0.46 |
| **HRV frequency-domain, per SD** | stβ [95% CI] |  | stβ [95% CI] |  | stβ [95% CI] |  | stβ [95% CI] |  | stβ [95% CI] |  |
| Model 4A, n=2,005 | **-0.05 (-0.09 to 0.00)** | **0.041** | -0.04 (-0.08 to 0.01) | 0.11 | -0.04 (-0.08 to 0.01) | 0.11 | -0.03 (-0.08 to 0.01) | 0.13 | -0.01 (-0.05 to 0.04) | 0.74 |
| Model 4B, n=1,676 | **-0.05 (-0.11 to -0.004)** | **0.034** | -0.04 (-0.08 to 0.01) | 0.144 | -0.04 (-0.09 to 0.01) | 0.14 | -0.04 (-0.09 to 0.01) | 0.14 | -0.004 (-0.05 to 0.05) | 0.87 |
| Model 4C, n=1,440 | **-0.07 (-0.12 to -0.01)** | **0.015** | -0.05 (-0.10 to 0.01) | 0.084 | -0.04 (-0.09 to 0.00) | 0.063 | -0.02 (-0.07 to 0.04) | 0.55 | 0.00 (-0.06 to 0.05) | 0.86 |
| Model 4D, n=2,007 | **-0.05 (-0.09 to 0.00)** | **0.039** | -0.04 (-0.08 to 0.01) | 0.10 | -0.04 (-0.08 to 0.01) | 0.093 | -0.03 (-0.08 to 0.01) | 0.14 | -0.01 (-0.05 to 0.04) | 0.74 |
| Model 4E. n=1,836 | **-0.05 (-0.10 to 0.00)** | **0.037** | -0.04 (-0.08 to 0.01) | 0.12 | -0.04 (-0.09 to 0.01) | 0.081 | -0.04 (-0.08 to 0.01) | 0.10 | -0.01 (-0.06 to 0.04) | 0.68 |
| Model 4F, n=1,836 | **-0.05 (-0.10 to 0.00)** | **0.033** | -0.04 (-0.09 to 0.00) | 0.079 | -0.04 (-0.09 to 0.00) | 0.079 | -0.04 (-0.09 to 0.00) | 0.063 | -0.04 (-0.09 to 0.01) | 0.093 |

Standardized regression coefficient (stβ) represents the difference in beta cell index in SD per SD lower HRV index.. Values per SD are numerically similar to the numbers reported in Table 1.

Bold indicates P-value<0.05.

Variables entered in the models in addition to HRV are (where appropriate): age, sex, and educational status (low, medium, high), Matsuda Index, office systolic blood pressure, total cholesterol/HDL cholesterol ratio, use of antihypertensive or lipid-modifying medication (yes/no), BMI, smoking status (current, ever, never), and alcohol consumption status (none, low, high).

Abbreviations: SD, standard deviation; CI, confidence interval; HRV, heart rate variability, HDL, high density lipid; BMI, body-mass index. All abbreviations for indices of HRV are presented in the Methods section.

Supplemental Table S11 Glucose metabolism status-stratified associations of time- and frequency- domain heart rate variability with C-peptidogenic index, overall insulin secretion, beta cell glucose sensitivity, beta cell potentiation factor, and beta cell rate sensitivity (model 3B)

|  | C-peptidogenic index | *P-value* | Overall insulin secretion | *P-value* | Beta cell glucose sensitivity | *P-value* | Beta cell potentiation factor | *P-value* | Beta cell rate sensitivity | *P-value* |
| --- | --- | --- | --- | --- | --- | --- | --- | --- | --- | --- |
| **HRV time- domain, per SD** | stβ [95% CI] |  | stβ [95% CI] |  | stβ [95% CI] |  | stβ [95% CI] |  | stβ [95% CI] |  |
| NGM, n=1,208 | -0.05 (-0.11; 0.01) | 0.11 | 0.01 (-0.04; 0.06) | 0.7 | 0.00 (-0.06; 0.06) | >0.9 | 0.01 (-0.05; 0.07) | 0.8 | 0.01 (-0.05; 0.07) | 0.8 |
| Prediabetes, n=323 | 0.06 (-0.06 to 0.17) | 0.33 | 0.08 (-0.03 to 0.18) | 0.14 | 0.11 (0.00 to 0.21) | 0.053 | **-0.18 (-0.29 to -0.06)** | **0.002** | **0.12 (0.01 to 0.23)** | **0.041** |
| Type 2 diabetes, n= 476 | -0.08 (-0.17 to 0.01) | 0.084 | -0.02 (-0.10 to 0.06) | 0.56 | -0.08 (-0.17 to 0.01) | 0.077 | -0.03 (-0.12 to 0.06) | 0.56 | -0.03 (-0.12 to 0.06) | 0.53 |
| **HRV frequency- domain, per SD** | stβ [95% CI] |  | stβ [95% CI] |  | stβ [95% CI] |  | stβ [95% CI] |  | stβ [95% CI] |  |
| NGM, n=1,208 | -0.05 (-0.11; 0.01) | 0.13 | 0.00 (-0.05; 0.06) | 0.9 | 0.00 (-0.06; 0.06) | >0.9 | 0.00 (-0.05; 0.06) | 0.9 | 0.01 (-0.05; 0.07) | 0.8 |
| Prediabetes, n=323 | 0.05 (-0.06 to 0.17) | 0.36 | 0.09 (-0.02 to 0.19) | 0.10 | **0.11 (0.00 to 0.22)** | **0.047** | **-0.17 (-0.28 to -0.05)** | **0.004** | **0.13 (0.02 to 0.25)** | **0.024** |
| Type 2 diabetes, n= 476 | -0.07 (-0.16 to 0.02) | 0.12 | -0.02 (-0.09 to 0.06) | 0.70 | -0.07 (-0.16 to 0.02) | 0.11 | -0.02 (-0.11 to 0.07) | 0.72 | -0.02 (-0.11 to 0.07) | 0.67 |

Standardized regression coefficient (stβ) represents the difference in beta cell index (in SD) per 1-SD lower HRV measure, where time domain HRV was estimated from SDNN, SDANN, RMSSD, SDNN index and pNN50; and frequency domain was estimated from TP, ULF, VLF, LF, and HF.

Bold indicates p<0.05.

Variables entered in model 3B in addition to HRV: age, sex, and educational status (low, medium, high), Matsuda Index; office systolic blood pressure, total cholesterol/HDL cholesterol ratio, use of antihypertensive or lipid-modifying medication (yes/no), BMI, smoking status (current, ever, never), and alcohol consumption status (none, low, high).

Abbreviations: SD, standard deviation; CI, confidence interval; HRV, heart rate variability, HDL, high density lipid; BMI, body-mass index. All abbreviations for indices of HRV are presented in the Methods section.

Supplemental Table S12 Glucose metabolism status-stratified associations of time- and frequency- domain heart rate variability with the beta cell composite score (model 3B)

|  |  | **Beta cell composite score, per SD** | |
| --- | --- | --- | --- |
|  | Glucose metabolism strata | stβ [95% CI] | *P-value* |
| **HRV time- domain composite score, per SD lower** | NGM, n=1,208 | -0.01 (-0.07; 0.05) | 0.79 |
|  | Prediabetes, n=323 | 0.07 (-0.05 to 0.18) | 0.25 |
|  | Type 2 diabetes, n= 476 | -0.06 (-0.15 to 0.02) | 0.15 |
| **HRV frequency- domain composite score , per SD lower** | NGM, n=1,208 | -0.01 (-0.07; 0.05) | 0.72 |
|  | Prediabetes, n=323 | 0.08 (-0.04 to 0.19) | 0.18 |
|  | Type 2 diabetes, n= 476 | -0.05 (-0.14 to 0.04) | 0.24 |

Standardized regression coefficient (stβ) represents the difference in beta cell composite score (in SD) per 1-SD lower time- or frequency-domain HRV composite score.

Bold indicates p<0.05.

Variables entered in model 3B in addition to HRV: age, sex, and educational status (low, medium, high), Matsuda Index; office systolic blood pressure, total cholesterol/HDL cholesterol ratio, use of antihypertensive or lipid-modifying medication (yes/no), BMI, smoking status (current, ever, never), and alcohol consumption status (none, low, high).

Abbreviations: SD, standard deviation; CI, confidence interval; HRV, heart rate variability, HDL, high density lipid; BMI, body-mass index. All abbreviations for indices of HRV are presented in the Methods section.

Supplemental Table S13 Associations of time- and frequency- domain heart rate variability with C-peptidogenic index, overall insulin secretion, beta cell glucose sensitivity, beta cell potentiation factor, and beta cell rate sensitivity in individuals with prediabetes, with adjustment for Matsuda Index (model 2A) or adjustment for total insulin secretion, estimated as C-peptide _AUC_ from the 7-point oral glucose tolerance test (model 2B)

|  | C-peptidogenic index | *P-value* | Overall insulin secretion | *P-value* | Beta cell glucose sensitivity | *P-value* | Beta cell potentiation factor | *P-value* | Beta cell rate sensitivity | *P-value* |
| --- | --- | --- | --- | --- | --- | --- | --- | --- | --- | --- |
| **HRV time-domain, per SD** | stβ [95% CI] |  | stβ [95% CI] |  | stβ [95% CI] |  | stβ [95% CI] |  | stβ [95% CI] |  |
| Crude | 0.04 (-0.07 to 0.15) | 0.50 | 0.13 (0.01 to 0.24) | **0.027** | 0.15 (0.04 to 0.26) | **0.008** | -0.17 (-0.29 to -0.06) | **0.002** | 0.11 (0.00 to 0.22) | 0.055 |
| Model 1 | 0.04 (-0.08 to 0.15) | 0.53 | 0.13 (0.02 to 0.25) | **0.027** | 0.14 (0.03 to 0.26) | **0.014** | -0.17 (-0.28 to -0.06) | **0.003** | 0.12 (0.01 to 0.24) | **0.032** |
| Model 2A | 0.04 (-0.08 to 0.15) | 0.52 | 0.08 (-0.02 to 0.18) | 0.13 | 0.11 (0.00 to 0.21) | **0.049** | -0.17 (-0.28 to -0.06) | **0.003** | 0.10 (-0.01 to 0.21) | 0.066 |
| Model 2B | 0.02 (-0.09 to 0.14) | 0.68 | 0.03 (-0.03 to 0.09) | 0.28 | 0.07 (-0.02 to 0.16) | 0.11 | -0.16 (-0.27 to -0.05) | **0.006** | 0.09 (-0.02 to 0.20) | 0.11 |
| **HRV frequency-domain, per SD** | stβ [95% CI] |  | stβ [95% CI] |  | stβ [95% CI] |  | stβ [95% CI] |  | stβ [95% CI] |  |
| Crude | 0.04 (-0.07 to 0.15) | 0.49 | 0.14 (0.02 to 0.25) | **0.017** | 0.16 (0.05 to 0.27) | **0.005** | -0.17 (-0.28 to -0.06) | **0.003** | 0.11 (0.00 to 0.23) | **0.048** |
| Model 1 | 0.04 (-0.08 to 0.15) | 0.51 | 0.14 (0.03 to 0.26) | **0.016** | 0.15 (0.04 to 0.27) | **0.009** | -0.16 (-0.28 to -0.05) | **0.005** | 0.13 (0.02 to 0.25) | **0.022** |
| Model 2A | 0.04 (-0.07 to 0.15) | 0.51 | 0.09 (-0.01 to 0.19) | 0.072 | 0.11 (0.01 to 0.22) | **0.039** | -0.16 (-0.27 to -0.05) | **0.004** | 0.12 (0.01 to 0.23) | **0.037** |
| Model 2B | 0.02 (-0.09 to 0.14) | 0.68 | 0.03 (-0.03 to 0.09) | 0.29 | 0.07 (-0.02 to 0.16) | 0.11 | -0.15 (-0.27 to -0.04) | **0.009** | 0.09 (-0.02 to 0.20) | 0.093 |

Standardized regression coefficient (stβ) represents the difference in beta cell index (in SD) per 1-SD lower HRV measure, where time domain HRV was estimated from SDNN, SDANN, RMSSD, SDNN index and pNN50; and frequency domain was estimated from TP, ULF, VLF, LF, and HF.

Bold P-value indicates p<0.05.

Variables entered in the models in addition to HRV: Crude: none; model 1: age, sex, and educational status (low, medium, high); model 2A: model 1 + Matsuda Index; model 2B: model 1 + total insulin secretion (estimated as C-peptide _AUC_ from the 7-point oral glucose tolerance test).

Abbreviations: SD, standard deviation; CI, confidence interval; HRV, heart rate variability. All abbreviations for indices of HRV are presented in the Methods section.
